# Scaling behaviour in music and cortical dynamics interplay to mediate music listening pleasure

**DOI:** 10.1038/s41598-019-54060-x

**Published:** 2019-11-27

**Authors:** Ana Filipa Teixeira Borges, Mona Irrmischer, Thomas Brockmeier, Dirk J. A. Smit, Huibert D. Mansvelder, Klaus Linkenkaer-Hansen

**Affiliations:** 1 Department of Integrative Neurophysiology, Center for Neurogenomics and Cognitive Research (CNCR), Amsterdam Neuroscience, VU Amsterdam, Amsterdam, 1081 HV Netherlands; 2 Psychiatry department, Amsterdam Neuroscience, Academic Medical Center, University of Amsterdam, 1081 HJ Amsterdam, The Netherlands

**Keywords:** Electrophysiology, Scale invariance, Cognitive neuroscience, Human behaviour, Statistical physics, thermodynamics and nonlinear dynamics

## Abstract

The pleasure of music listening regulates daily behaviour and promotes rehabilitation in healthcare. Human behaviour emerges from the modulation of spontaneous timely coordinated neuronal networks. Too little is known about the physical properties and neurophysiological underpinnings of music to understand its perception, its health benefit and to deploy personalized or standardized music-therapy. Prior studies revealed how macroscopic neuronal and music patterns scale with frequency according to a 1/*f*^*α*^ relationship, where a is the scaling exponent. Here, we examine how this hallmark in music and neuronal dynamics relate to pleasure. Using electroencephalography, electrocardiography and behavioural data in healthy subjects, we show that music listening decreases the scaling exponent of neuronal activity and—in temporal areas—this change is linked to pleasure. Default-state scaling exponents of the most pleased individuals were higher and approached those found in music loudness fluctuations. Furthermore, the scaling in selective regions and timescales and the average heart rate were largely proportional to the scaling of the melody. The scaling behaviour of heartbeat and neuronal fluctuations were associated during music listening. Our results point to a 1/*f* *resonance* between brain and music and a temporal rescaling of neuronal activity in the temporal cortex as mechanisms underlying music appreciation.

## Introduction

Music is a cross-cultural phenomenon, and biological constraints underlie its appeal^1^. Music listening recruits the concerted activity of several cortical and subcortical regions^2,3^; its emotive power relies on extensive neuronal circuits^4^ and modulates neurochemistry^5^. Besides being often used to regulate emotions^6^, music listening appears valuable in, e.g., cognitive rehabilitation of post-stroke and dementia patients^7,8^, diagnosis of disorders of consciousness^9^, and visual-spatial reasoning^10^. Moreover, pleasant music boosts problem-solving skills^11,12^. However, what makes music pleasurable and why it emerges as widely beneficial in cognition remains unclear.

Music theorists argue that the enjoyment of music derives from how the music unfolds in time. A balance between regularity and variation in its composition^13^ and individual innate and learned expectations^14^ shape the emotions evoked. Fractal theory is a notable way of conceptualising this balance of predictability and surprise within the music flow. Random fractals encompass irregular fluctuations that show a statistical resemblance at several timescales and are therefore called self-similar^15,16^. The degree of self-similarity can be quantified by a scaling exponent (*α*), which captures the relationship between the averaged fluctuation, *F*(*t*), and the timescale, *t*: *F*(*t*) ~ *t*^*α*^. When 0.5 < *α* < 1, there are persistent long-range temporal correlations present; *α* = 1 (1/*f* noise) represents a compromise between the unpredictable randomness of white noise (*α* = 0.5) and the smoothness of Brownian noise (*α* = 1.5)^17^. A straightforward relationship with the frequency-domain is such that the spectrum *S*(*f*) displays an inverse power-law scaling (*S*(*f*) ~ 1/*f*^*β*^, where *β* = 2*α* − 1). Musical pitch and loudness^18,19^ and musical rhythms^20,21^ obey approximately a 1/*f* power-law distribution and, fractal properties differentiate composers and genres^20,22^. Compositions in which the frequency and duration of the notes follow 1/*f* distributions sound more pleasing than 1/*f*^2^ or random ones^23^ and 1/*f* deviations in computer-generated beats humanise and create more pleasant rhythms than random deviations^21^. Scaling behaviour is also ubiquitous in multi-level neuronal systems. In particular, neurons from earlier stages of the auditory pathways are tuned to sounds with different 1/*f*^*β*^ spectra, while neurons in the auditory cortex favour 1/*f* statistics^24^. Fluctuations in neuroelectric and neuromagnetic brain activity display 1/*f*^*β*^ scaling under rest^25–28^ and music^29^. Self-similarity further characterises the heartbeat variations^30^ and such dynamical organization of the nervous system is functionally relevant^17,31–33^. Humans can apprehend recursive fractal rules embedded in tone sequences^34^, predict 1/*f* better than random tempo fluctuations^35^ and, a preferential cortical tracking of tones occurs when its pitches display long-range temporal correlations^36^. Furthermore, electrophysiological evidence suggests humans process long-distance dependencies typical of music syntax^37^. Altogether, it led us to hypothesise that the scaling of music shapes the neuronal scaling behaviour during listening and to posit that the brain’s sensitivity to music—and the pleasure derived from listening—lies in their shared similar dynamical complex properties^38^.

**Figure 1:**
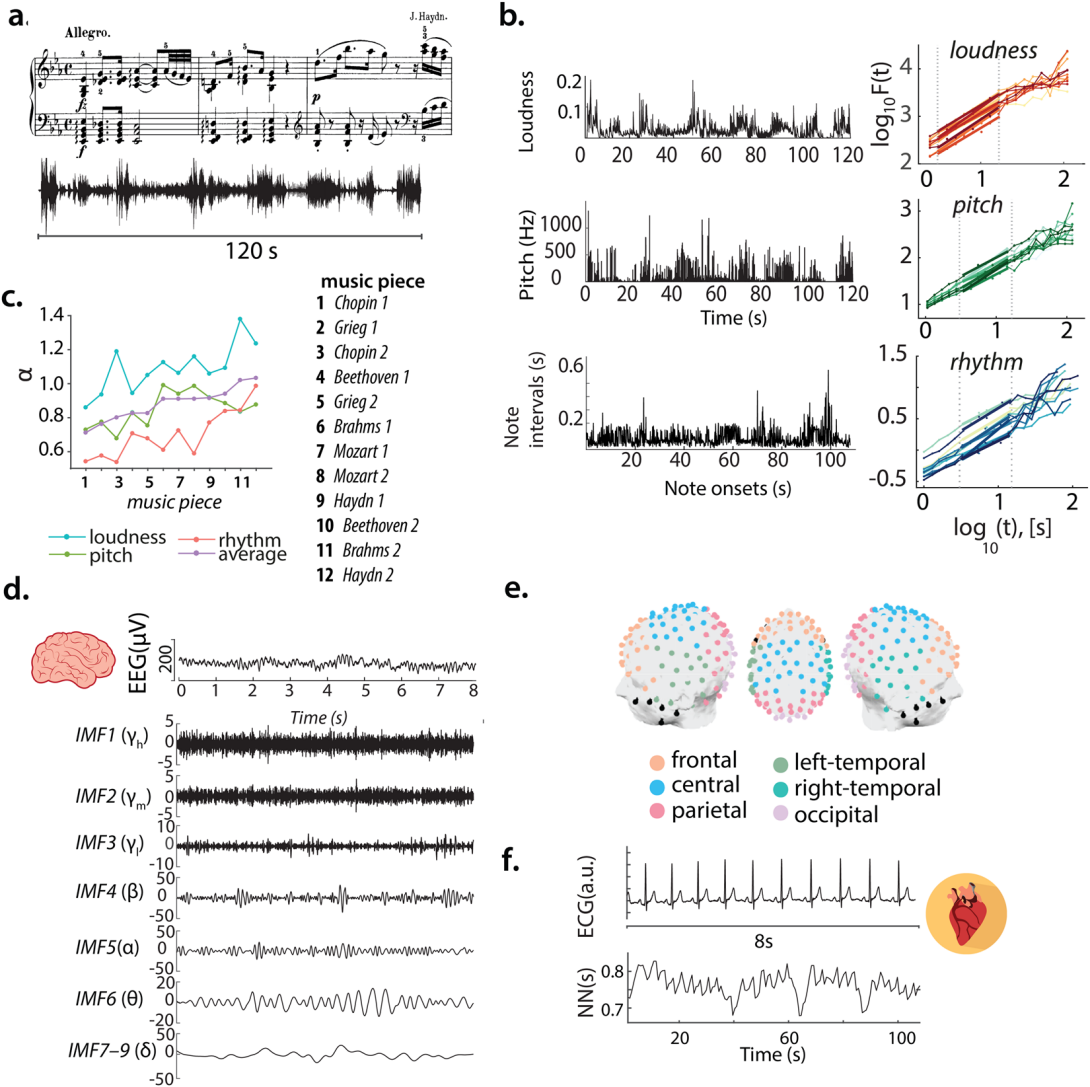
Scheme of the investigation. (**a**) Stimuli example—excerpt of the sound signal from the Sonata no. 62 Allegro (Haydn) and partial score. (**b**) (*Left*) Correspondent loudness, pitch and rhythm time series representing respectively the audio envelope, successive dominant note-frequency changes and note intervals. (*Right*) Approximate linear relationship between log *t* and the average fluctuation log *F*(*t*) for *t* ∈ [3, 15]*s* reveals a fractal scaling characteristic of how the musical features unfold in time. (**c**) Scaling exponents obtained for each of the music dimensions form a gradient from near randomness (0.5) to smooth and highly correlated fluctuations (>1). (**d**) Broadband EEG trace and the different timescales (Empirical Mode Decomposition) analysed. (**e**) Channels (dots) and regions (colour-coded) analysed (cf. Methods for details and Table 1 for a glossary of the main experiment variables). (**f**) Heartbeat and interbeat intervals (NN) obtained from ECG signals. Music sheet is a courtesy of Musopen. Brain and heart images in (**d**,**f**) by Sinisa Maric and Marcus Hartmann, under Pixabay license.

Here, we characterise the self-similarity of fluctuations in loudness, pitch, and rhythm of 12 classical pieces (Fig. 1b,c) and analyse the scaling behaviour of multiscale neuronal activity from different scalp regions (Fig. 1d,e) and cardiac interbeat intervals (Fig. 1f) of healthy individuals—at baseline and during music listening—and associate self-reported pleasure with these measures (see also Table 1).

**Table 1:** Glossary with the main experimental variables and its (multi)-level descriptions.

Variable	Description	Multi-level description
*α*_*brain*_	scaling exponent, indicating the persistence or self-similarity in modulations of neuronal activity (see Fig. 1d for components studied: ${\alpha }_{{\gamma }_{h}}$, ${\alpha }_{{\gamma }_{m}}$, ${\alpha }_{{\gamma }_{l}}$, *α*_*β*_, *α*_*α*_, *α*_*θ*_ and *α*_*δ*_)	${\bar{\alpha }}_{individual}$ (averaged values across pieces for each subject)
		${\bar{\alpha }}_{piece}$ (averaged values across subjects for each piece)
*α*_*music*_	scaling exponent characterising the self-similarity of a musical feature (see Fig. 1b for musical features studied: *α*_*loudness*_, *α*_*pitch*_, *α*_*rhythm*_, *α*_*average*_)	
*s*	behavioural scores (ratings of pleasure, concentration and familiarity)	${\bar{s}}_{individual}$ (averaged values across pieces for each subject)
		${\bar{s}}_{piece}$ (averaged values across subjects for each piece)
*α*_1_ (heart)	scaling behaviour exponent of short-term heart rate variability	
AVNN (heart)	average of normal sinus interbeat intervals	
1/*f* noise	stochastic process which displays a power spectral density, *S*(*f*), with the form *S*(*f*) = 1/*f*^*β*^, where *f* is the frequency. The *β* exponent is related to the *colour* of the signal, that is to the degree the signal is autocorrelated, a measure of self-similar dynamics. If *β* = 0, the power spectrum is flat and there are no autocorrelations present (white noise), if *β* = 2, the signal is highly autocorrelated corresponding to integrated white noise (i.e., Brownian or red noise). The special case when *β* = 1 yields a signal with moderate autocorrelations, often referred as pink noise. In this Paper, 1/*f* refers to pink noise and 1/*f*^*β*^ to other generic cases of 1/*f* scaling.	
1/*f* resonance	phenomenon by which a stimulus with 1/*f* variability transmits maximal information to another system if the latter is close to the ideal 1/*f*-noise condition	

## Results

### Neuronal scaling behaviour during music listening

**Figure 2:**
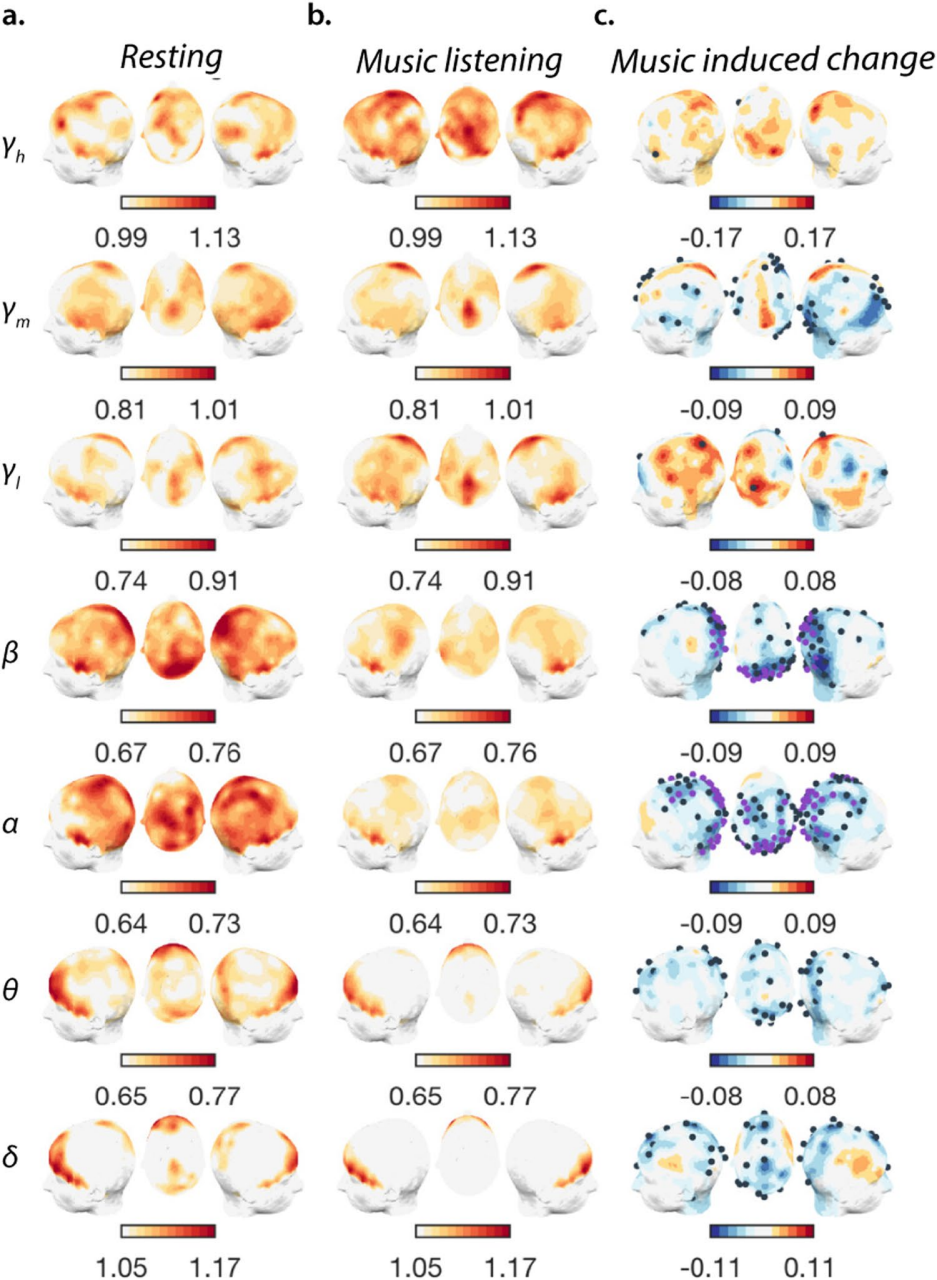
Music-induced decrease in the scaling of the envelope fluctuations in most frequency ranges relative to the rest, default-state. Head surface maps of the scaling exponent of neuronal components (*γ*_*h*_ − *δ*) during rest (**a**) during a music listening task (**b**) and of the difference between the latter two. (**c)** The decreases are accentuated in the parietal and occipital regions. Channels marked with dark blue dots display significant differences (*p* < 0.05, uncorrected), purple dots signal significant differences after FDR correction (*q* = 0.05), minimum *p* = 0.005 (*α*), *p* = 0.011 (*β*)).

Twenty-eight participants underwent a music-listening task after a baseline period of eyes-closed rest. We quantified the degree of self-similarity (*α*) in seven neuronal components (Fig. 1d; *Methods*). Music listening decreased the *α* value of most frequency-dependent levels relative to the resting-state (Fig. 2). These decreases were conspicuous in the parietal and occipital regions of neuronal activity correspondent to the *α*- (${\bar{z}}_{parietal}=-\,2.50$, ${\bar{r}}_{parietal}=0.33$; ${\bar{z}}_{occipital}=-\,2.52$, ${\bar{r}}_{occipital}=0.34$, *p*_*c*_ < 0.005; Wilcoxon signed-rank test: *z*—z-score, *r*—effect size), *β*- (${\bar{z}}_{parietal}=-\,2.41$, ${\bar{r}}_{parietal}=0.32$; ${\bar{z}}_{occipital}=-\,2.14$, ${\bar{r}}_{occipital}=0.29$, *p*_*c*_ < 0.011) and *γ*_*m*_-components (${\bar{z}}_{parietal}=-\,1.26$, ${\bar{r}}_{parietal}=0.17$; ${\bar{z}}_{occipital}=-\,1.12$, ${\bar{r}}_{occipital}=0.15$, *p* < 0.048; n.s. differences after FDR correction). Additional significant decreases occurred in the central area exclusively in the *α*-component (${\bar{z}}_{central}=-\,2.06$, ${\bar{r}}_{central}=0.27$, *p* < 0.048) and in the right-frontal channels within the *γ*_*m*_ neuronal component (${\bar{z}}_{frontal}=-\,1.55$, ${\bar{r}}_{frontal}=0.21$, *p* < 0.043) (Fig. 2c). On a global topographic level, the median scaling of neuronal oscillations also decreased but only in activity below *γ* (Supplementary Fig. 2, SI Results). The scaling behaviour characterises how the amplitude envelope of selected frequency components is modulated on different time scales; the fact that this property did not remain static across states suggests that it may capture meaningful functional processes. Of note, this music-induced change of neuronal assembly dynamics is distinct from changes in spectral power. In fact, average spectral power increased (*α* − *β*), decreased (*θ*) or remained unchanged in the *γ* amplitude fluctuations (Supplementary Fig. 3).

### Music-induced behaviour

**Figure 3:**
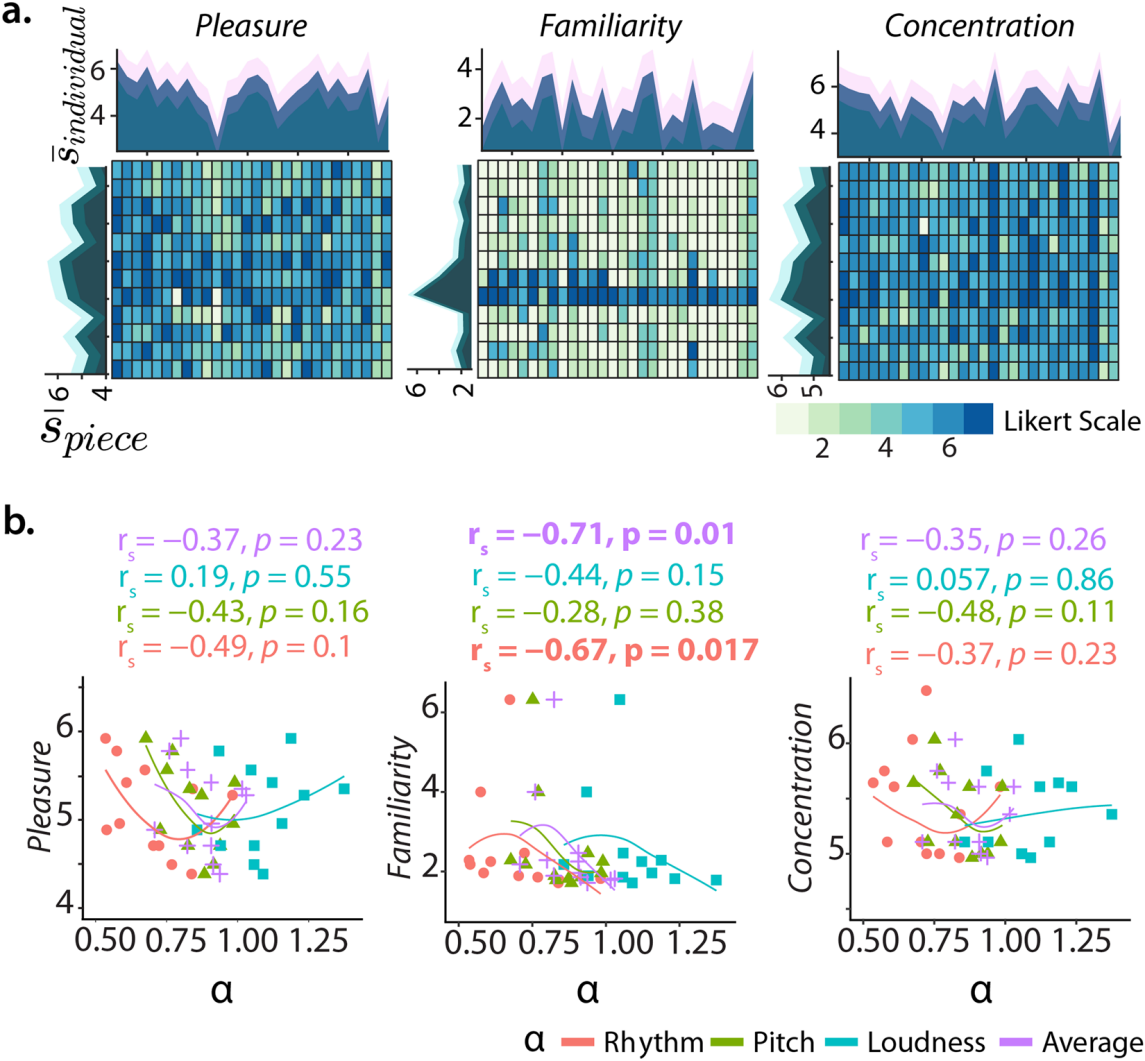
Behaviour induced by music listening and its relationship to musical features. (**a**) Individual pleasure, familiarity and concentration ratings for each participant (*x-axis*) and piece (*y-axis*) and their average ($\bar{s}$) per individual/piece (dark colour) and SD (shade in light colours) (lateral plots). (**b**) Relationship between ${\bar{s}}_{piece}$ and the scaling exponent (*α*_*music*_) of each piece for all music dimensions; only ${\bar{s}}_{piece}$ of Familiarity shows a significant association with the dynamics of the stimuli musical dimensions.

On average, individuals agreed with experiencing Pleasure during music listening (${\bar{s}}_{individual}=5.1\pm 0.7$, mean ± SD) and being focused on the listening (5.4 ± 0.7) whereas, Familiarity was most often rated with low scores (2.5 ± 0.9), with only two pieces being widely familiar (Fig. 3a). There was considerable individual variability in which pieces elicited more pleasure (Krippendorff’s *α* = 0.12), were more familiar (*α* = 0.27) or elicited more focus (*α* = 0.07) (see also ${\bar{s}}_{piece}$ Fig. 3a). Familiarity was inversely correlated to the scaling of the musical rhythm (*α*_*rhythm*_; *r*_*s*_ = −0.71, *p* = 0.01) and the averaged scaling of all features (*α*_*average*_; *r*_*s*_ = −0.67, *p* = 0.02) (Fig. 3b). On average, the level of Pleasure experienced was strongly associated with the Concentration ratings of each piece (*r*_*s*_ = −0.71, *p* = 4.2 . 10^−2^) and participant (*r*_*s*_ = 0.73, *p* = 1.1.  10^−5^). However, while overall Pleasure/Concentration elicited by a piece positively correlated with Familiarity (*r*_*s*_ = 0.58, *p* = 0.05; Supplementary Fig. 4a), participants experiencing more pleasure had lower familiarity scores (*r*_*s*_ = −0.36, *p* = 0.06) and more concentrated participants had higher familiarity scores (*r*_*s*_ = −0.55, *p* = 0.0025; Supplementary Fig. 4b). Thus, for the music stimuli used in this experiment, the variance in Familiarity did not explain a great percentage of the variance in Pleasure and a lower individual Familiarity appears linked to higher concentration.

### Neuronal scaling behaviour links to individual musical pleasure

**Figure 4:**
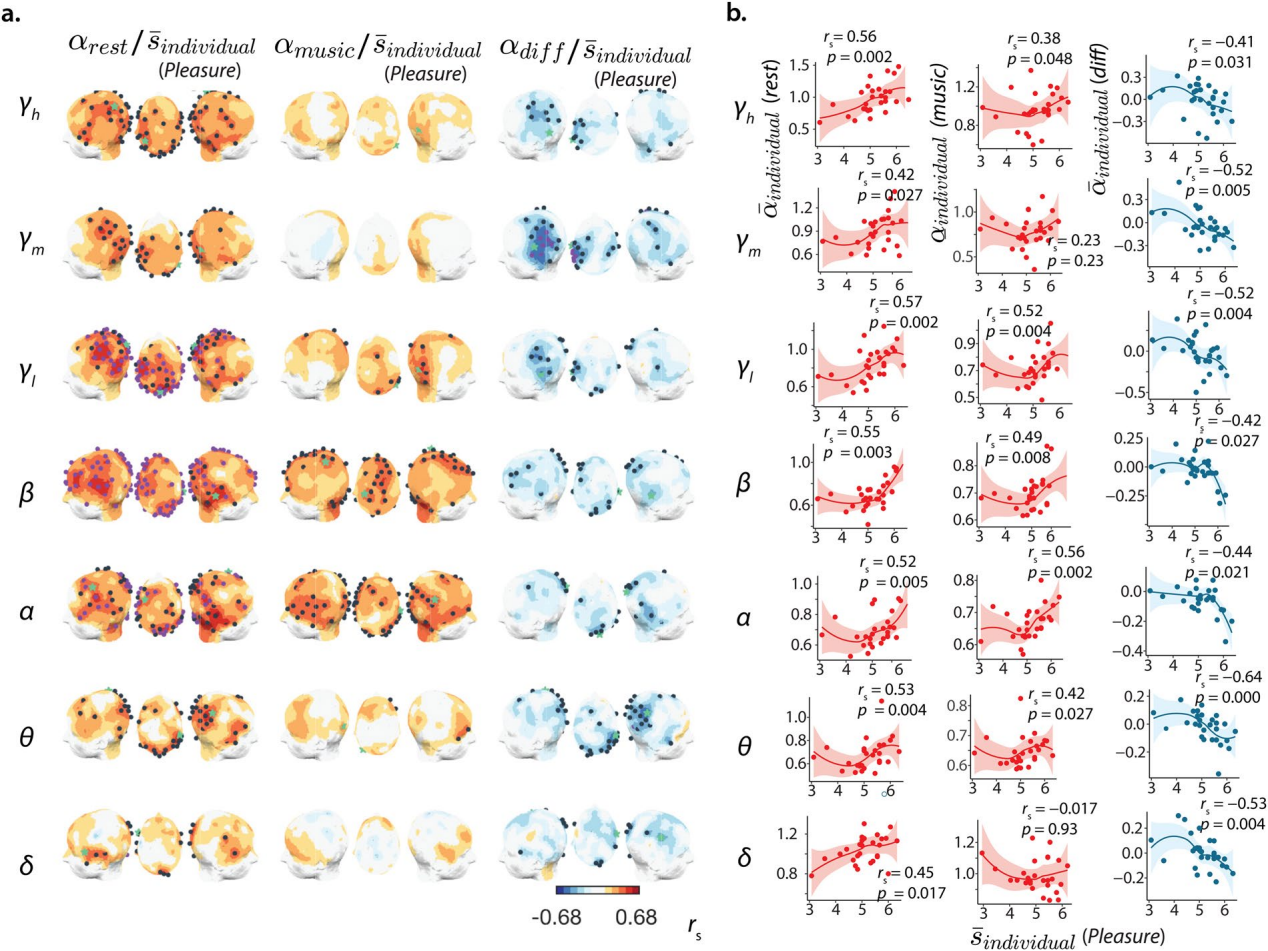
The scaling behaviour of neuronal activity during baseline and its induced change during listening capture the individual pleasure experienced with the music. (**a**) Head surface mappings of the associations between Pleasure (${\bar{s}}_{individual}$) and the scaling exponents of the components (*γ*_*h*_ − *δ*) during baseline (rest), during a music listening task, and with the induced change in scaling between the latter. The channels marked in dark blue indicate a nominally significant correlation (Spearman coefficient *r*_*s*_, *p* < 0.05), the channels in purple show significant association after FDR correction (*q* = 0.1, minimum *p*-values at this FDR spanned between 2.82 × 10^−4^ and 0.025). (**b**) Scatter plots of the highlighted channels (green star in (**c**) exemplify the individual values (*n* = 28), a locally weighted regression line was added to aid visualising the relationship and the shadowed area represent the confidence interval.

It is generally accepted that the enjoyment of music is subjective, conditioned by taste and personal history. We found that the self-similarity of the individuals’ ongoing neuronal activity correlates with the pleasure experienced during music listening (Fig. 4a). Specifically, individuals exhibiting scaling exponents of the amplitude fluctuations of resting-state neuronal activity with higher values, close to 1, were more likely to enjoy the music (Fig. 4b). This pattern of association was general, however, it occurred most significantly in the *α*-, *β*- and *γ*_*l*_-components. The scaling of amplitude modulations of the music (*α*_*loudness*_) was also roughly 1 in the group of music pieces used in the experiment (*α*_*loudness*_ = 1.07 ± 0.14, Fig. 1c). The regions constituting loci with the most predictive power of overall music enjoyment were extended throughout the cortex, in particular in the parietal (${\bar{r}}_{s}(26)=0.37(\alpha )$; 0.34(*β*); 0.41(*γ*_*l*_)), occipital (${\bar{r}}_{s}(26)=0.34(\alpha )$; 0.40(*β*); 0.33(*γ*_*l*_)) and temporal lobes (${\bar{r}}_{s}(26)=0.36(\alpha )$; 0.37(*β*); 0.34(*γ*_*l*_)) (*r*_*s*_, Spearman coefficient; *q* = 0.1, FDR). During music listening, a similar coarse profile characterised this association albeit less significantly (*ns*. for *q* = 0.1, FDR) and with inferior magnitude, mainly in the temporal cortices (${\bar{r}}_{s}(26)=0.37(\alpha )$; 0.24(*β*); 0.14(*γ*_*l*_)). Strikingly, individual differences in the scaling behaviour (*music-rest*) were inversely associated with perceived pleasure, i.e., the highest music-induced decreases in neuronal scaling exponents were likely to occur for the participants who scored high on Pleasure. The greatest proportions of shared variance were, in this case, centred in the *γ*_*m*_ activity of the temporal and, less significantly, in the parietal cortices. Particularly, in the left-temporal (${\bar{r}}_{s}(26)=-\,0.27({\gamma }_{h})$; −0.42(*γ*_*m*_); −0.28(*γ*_*l*_)) as opposed to the right-temporal region (${\bar{r}}_{s}(26)=-\,0.20({\gamma }_{h})$; −0.24(*γ*_*m*_); −0.18(*γ*_*l*_)); (see Fig. 4a,b for statistical significance). Considering how pleasure and concentration ratings were strongly correlated (Supplementary Fig. 4), it is plausible that the *α*_*brain*_–Pleasure associations are merely related to concentration. To rule out this possibility, we also correlated the neuronal scaling exponents to the Concentration scores (Supplementary Fig. 5). Although topographically the associations resemble those found for Pleasure, the magnitude is diminished and significant interactions are scarce, suggesting the association to Pleasure is not primarily driven by a higher individual capacity to attend to the music.

### Interactions between neuronal, music and cardiac dynamics

**Figure 5:**
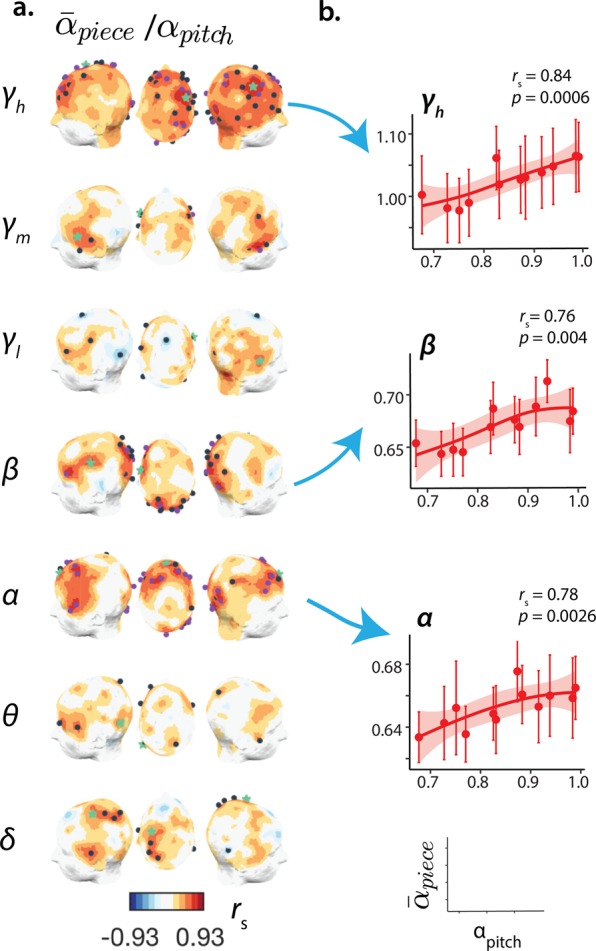
Correlation analysis reveals a link between the scaling behaviour of the music and the scaling of neuronal activity in the *α*, *β and γ*_*h*_-components. (**a**) Headplots of the correlation between the scaling exponent of the pitch series (*α*_*pitch*_) and the average scaling exhibited by the multiscale neuronal activity (${\bar{\alpha }}_{piece}$ for *γ*_*h*_ − *δ*) for the music pieces; the dark blue dots indicate a channel with significant correlation (Spearman coefficient *r*_*s*_, *p* < 0.05), the purple ones show significant association after FDR correction (*q* = 0.2, minimum *p* = 0.017 (*γ*_*h*_, *α*) and *p* = 0.006 (*β*)). (**b**) Scatterplots portray the association between the scaling of neuronal activity in the *γ*_*h*_, *β* and *α* components, for the channels highlighted with a green star in (**a**) and the scaling of pitch successions; the error bars denote the standard error of the mean.

**Figure 6:**
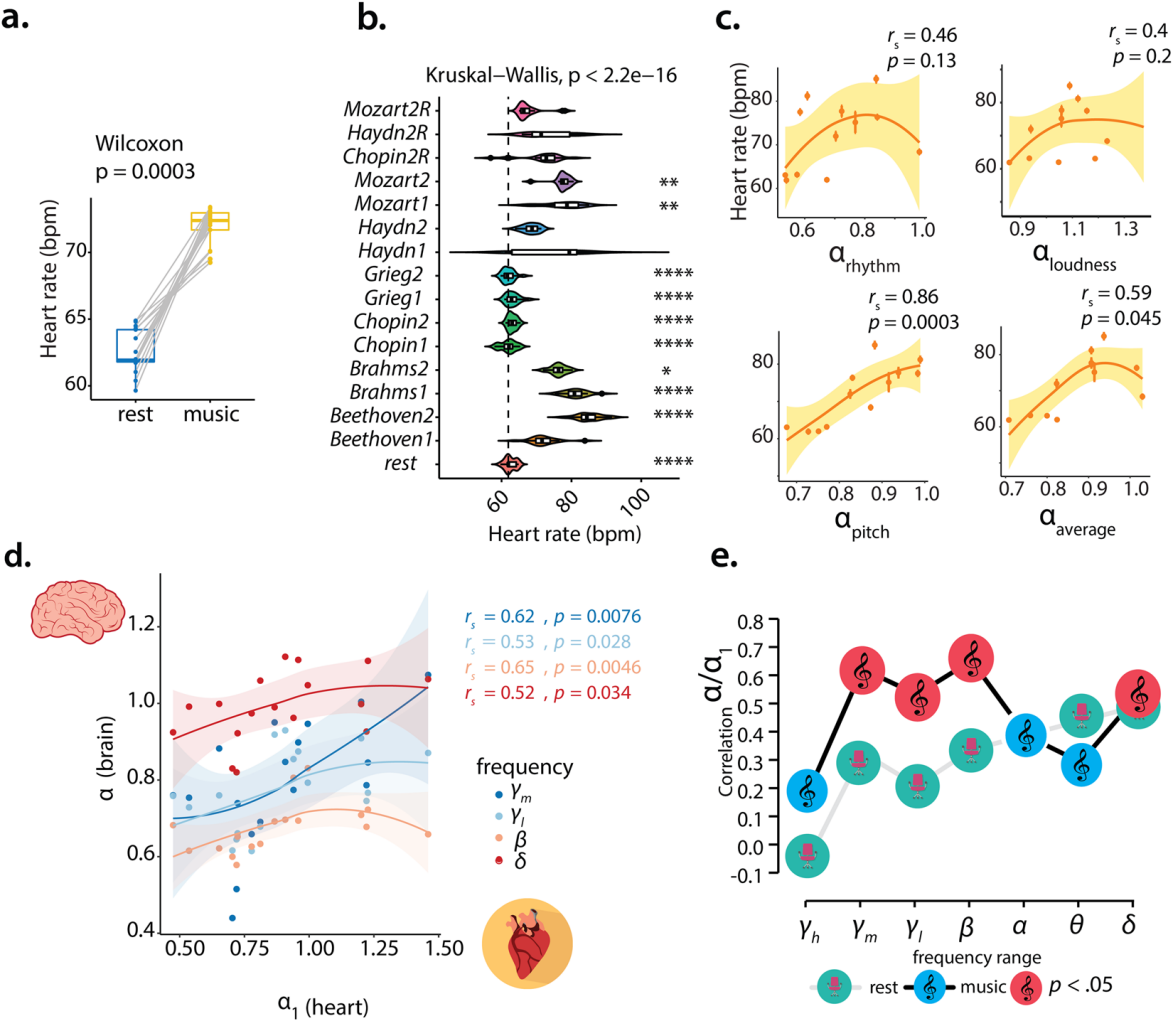
Heart rate dependency on musical dimensions of the music stimuli and 1/*f* resonance between brain and heart dynamics. (**a**) Average heart rate (AVNN) during music listening shows a significant increase relative to baseline. (**b**) Individual AVNN depends on music piece as shown by Violin plots with overlaid boxplots, the box limits show the 25^th^ and 75^th^ percentile together with the medians, whiskers extend 1.5 times the interquartile range (IQR) and the coloured polygons representing density estimates of AVNN. (**c**) Relationship between the scaling behaviour of the musical dimensions and the average AVNN for each piece; error bars indicate standard error of the mean. (**d**) The self-similarity in individual heart rate variability (*α*_1_) is associated with the neuronal scaling of both high- (*γ*_*h*_, *γ*_1_ and *β* activity) and low-frequency oscillations (*δ*). (**e**) Correlation between brain and *α*_1_ is strengthened during music relative to baseline suggesting music may facilitate an 1/*f* resonance between brain and heart. Brain and heart images in (**d**) by Sinisa Maric and Marcus Hartmann. Clef and chair images in (**e**) by rawpixel and Pettycon. All under Pixabay license.

We also sought to investigate the extent to which the temporal structure of the music signals determined the scaling of neuronal activity during music. By mapping the correlation between the scaling of different music features (*α*_*loudness*,*pitch*,*rhythm*_) and the average inter-subject neuronal scaling exponent for each piece (${\bar{\alpha }}_{piece}$), we found a significant association between the self-similarity of pitch successions and that of *γ*_*h*_-, *β*- and *α*-component dynamics in the occipital region (${\bar{r}}_{s}(10)=0.49({\gamma }_{h})$, *p*_*c*_ < 0.017; ${\bar{r}}_{s}(10)=0.45(\beta )$, *p*_*c*_ < 0.006; ${\bar{r}}_{s}(10)=0.51(\alpha )$, *p*_*c*_ < 0.017, FDR—*q* = 0.2) (Fig. 5). Some focal frontal and right-temporal locations had strong associations (Fig. 5a,b), and some of these were robust to a false-discovery rate correction at 10 and 5%. Rhythm and loudness showed a magnitude-wise similar positive correlation but with relative sparsity. Only a few clusters of the interactions ${\bar{\alpha }}_{piece}({\gamma }_{h})-{\alpha }_{rhythm}$ and ${\bar{\alpha }}_{piece}(\theta )-{\alpha }_{loudness}$ reached statistical significance after correction (Supplementary Fig. 7). We further probed the music effect on cardiac dynamics, using a subset of participants (*n* = 17) we found that average heart rate (AVNN) significantly increased during music (Fig. 6a), nevertheless AVNN values varied widely across pieces (Fig. 6b). Similarly to the neuronal effect, AVNN increased almost linearly with *α*_*pitch*_ (Fig. 6c); the summary of the dimensions (*α*_*average*_) also correlated significantly to AVNN despite non-strictly monotonic associations of this latter with *α*_*rhythm*/*loudness*_ and no relationship with the tempi of the pieces (Supplementary Fig. 8). Despite modulating the sinus rhythm and other standard heart-rate variability measures (Supplementary Fig. 9), music did neither consistently modulate the scaling of the interbeat intervals (*α*_1_) (Supplementary Fig. 10a), nor the scaling of the music associated with *α*_1_ (Supplementary Fig. 10b). Lastly, we investigated whether a correspondence between neuronal and cardiac dynamics could mediate the experience of music listening. To address this question, we correlated *α*_1_ to the global (average of channels) neuronal scaling exponents $[{\alpha }_{{\gamma }_{h}},{\alpha }_{{\gamma }_{m}},{\alpha }_{{\gamma }_{l}},\ldots ]$. We observed a significant positive correlation selectively within the *γ*_*h*_ − *β* and *δ* frequency-ranges (Fig. 6d); this association with the midhigh-frequency neuronal dynamics was significant and stronger during music vs. rest (Fig. 6e). Thus, we conclude that, during music listening, a synergistic dynamical interplay of heart rate and neuronal activity may be facilitated.

## Discussion

Embodied cognition emerged as the science explaining cognitive processes through the continuous interaction of bodies, brains and environment^39^. Within this framework, we have used a dynamical property—the scaling behaviour—to characterise how neuronal, cardiac activity and music unfold in time. We hypothesised that the pleasure of music mutually depends on the scaling behaviour of neuronal and musical features. This possibility deepens our knowledge of how the brain harnesses and is shaped by the dynamics of music, sheds light on the neuronal mechanisms underlying musical pleasure and may guide the deployment of music therapy. To this end, we analysed the EEG of individuals that listened to classical pieces characterised by different 1/*f* scaling. We report how music reshapes neuronal activity fluctuations over many seconds. The music-induced change in the scaling behaviour of these fluctuations and its baseline level associate with the pleasure experienced. These associations with pleasure were not caused by concentration or familiarity levels. In addition, the neuronal scaling behaviour covaries with the scaling of music, and an interdependence between the scaling of neuronal and cardiac dynamics emerges during music listening.

The decrease in the scaling exponent of neuronal activity (*α*_*brain*_) found during music aligns with the emerging view that tasks reduce the self-similarity in the ongoing fluctuations of brain field potentials^40^. These decreases may stem from merely eye-opening^41^, to greater attentional demands such as during auditory^42^ and visual^43^ tasks. Individuals who finger-tap more accurately to a fixed rhythm were also shown to have lower scaling exponents within the *α*-band^44^, which altogether with our results suggests that this temporal reorganisation is linked to tracking basic or complex rhythms. Our study consistently implicated the *α*, *β* and *γ* bands in music processing. Attending to auditory stimuli is long known to modulate *α* oscillations in the parieto-occipital region^45^, and the individual and music-specific changes observed in *α*_*brain*_ of the *α*-component converge with evidence on how music modulates parieto-occipital alpha power in an individual- and stimulus-specific fashion^46^. Further, the *β* − *γ* modulations are consistent with these neuronal bands substantiating predictive timing mechanisms of beat-processing^47^. The music-induced decreases of *α*_*brain*_ seemingly reflect a dynamical reorganisation of neuronal ensembles, known to resemble more closely 1/*f* noise (*α* = 1) during rest^48^, to adapt to the rapid demands underlying processing of stimuli by forming more transient but persistent spatiotemporal patterns. It may appear paradoxical that both the most pronounced music-induced decreases and the highest resting-state scaling exponents are linked to pleasure. Yet, *α*_*brain*_ during rest was positively correlated to pleasure in wide areas of the brain and the reductions in *α*_*brain*_ were focal to the (particularly left) temporal cortices. Hence, the spatial topography suggests that the decreases in *α*_*brain*_ are a proxy of the auditory processing, integrating the encoding of meaning from sounds^49^ and abstract representation of music^50^. The selective involvement of *γ*_*m*_ is also consistent with the role of >40 Hz activity in indexing task-specific processes during auditory perception^51^. This result supports a current theory of how the aesthetic musical emotions are linked to the satisfaction of a knowledge instinct^52,53^. In a nutshell, this theory proposes that pleasant music can overcome cognitive dissonance by allowing multiple contradictory beliefs to be reconciled, generating a synthesis of newer abstract meanings. Given that a decrease in self-similarity is arguably linked to an increased dimensional complexity^54^, we hypothesise that the pleasure induced decreases in *α*_*brain*_ exclusively in the left-temporal area—an area thought to harbour abstract neural representations—reflect a more flexible dynamical organisation of the underlying neuronal ensembles which may facilitate the embodiment of abstract concepts^55^. Conversely, the association of musical pleasure to *α*_*brain*_ during baseline reflects intrinsic traits constraining the listening experience.

Remarkably, the baseline values of individual *α*_*brain*_ from subjects that experienced more overall pleasure were close to 1 in the components most significantly associated with pleasure (viz., *α* − *γ*_*l*_). On average, the scaling of amplitude modulations of music (*α*_*loudness*_) was also 1. A 1/*f* scaling endows a system with the sensitivity to perturbations with a wide dynamic range, i.e., information at many timescales^56^ and, interacting systems which have matching 1/*f* scaling are thought to display maximal information exchange^38,57,58^. Thus, our results are consistent with the hypothesis that music exerts a strong influence on the brain by means of a 1/*f* resonance mechanism. This concept is akin to entrainment but differs from the common use of the latter (rhythmic processes interacting until eventually “locking-in” to a common phase). It can be thought as a derivation of the stochastic resonance phenomenon for the perturbation of a system by another system when they both have broadband dynamics with complex fractal signatures^59^. We have previously shown the importance of a similar phenomenon for speech comprehension^60^ and demonstrate here its relevance for music’s enjoyment. On another level, the relative distribution of low-frequency power has been linked to personality traits such as neuroticism and openness^61^, known to predispose music-induced emotions^62^—suggesting another facet of how intrinsic 1/*f* scaling connects to music’s pleasure.

Moreover, the positive correlation *α*_*pitch*_/*α*_*brain*_ extends previous findings of how neuromagnetic 41.5 Hz phase tracks the statistics of artificial tone sequences^36^. However, in contrast to this study where energy modulations were independent of the dynamics of the auditory stimulus, we unveiled a functional relationship between the scaling of pitch and *γ*_*h*_ fluctuations (as in *α*/*β*) during music listening in frontal and occipitoparietal networks. The foremost correlations (0.9) were located in the right frontotemporal region, concurring with the critical role of this region in melodic processing^63^ and in processing non-local dependencies in musical motifs^64^. However, prior research also found an inverse relationship between the scaling of pitch fluctuations and the scaling of neuronal activity (*α*-component) in the occipital area^65^. A possible reason for this discrepancy is the use of artificial 1/*f* tone sequences instead of natural music, like in the current paradigm. Notwithstanding a lack of effect of *α*_*pitch*_ on the scaling of heart rate (*α*_1_), the AVNN increased monotonically with *α*_*pitch*_. This discovery expands our knowledge of how the autonomic nervous system (ANS) is regulated by music’s temporal properties. Tempo has been often taken as the first proxy of music rhythm, yet both this paradigm and previous studies^66–68^ have revealed a nuanced role of tempo in shaping the AVNN of the listener. Our finding suggests that the scaling of music carries a predictive value when inferring ANS function not directly available from tempo. The selective correlation between the scaling exponent of both cortical *δ*, *β* and *γ*_*l*_/*γ*_*m*_ activity and cardiac dynamics suggests a pivotal role of these frequency channels in mediating communication between heart and brain systems. Interestingly, during rest, the scaling of neuromagnetic modulations around 3 (*δ*) and 30 (*β*) Hz is positively correlated to the scaling of HRV^69^, and *β* oscillations were identified as the primary hub mediating bidirectional brain-heart information transfer during sleep^70^. Since these studies were limited to frequencies below 30 Hz and distinct states, it remains to elucidate whether the observed relationship with the neuronal scaling of *γ* activity reflects a generalised means of interaction or if it is peculiar to music listening. To the best of our knowledge, no other studies concomitantly assessed *γ* and HRV, hindering any conclusion about the genericity of this mechanism. Conversely, although the interactive pattern pervades rest and music, the significance and magnitude of the interaction in *β* − *γ*_*m*_ was selectively enhanced during music. One possible root for this interchange is the fact that respiratory rate changes during music listening^71^; *α*_1_ (heart) reflects interbeat interval fluctuations which are dominated by the regular oscillations of respiration^30^ and could, therefore, be a source of the music-induced interactive changes. This hypothesis is further supported by recent iEEG evidence showing how the breathing cycle tracks power (40–150 Hz) in diverse cortical and limbic areas^72^.

Some limitations of this experiment limit the conclusions, namely, since we optimised the scaling gradient of the stimuli by first considering *α*_*pitch*_/*α*_*rhythm*_, we cannot rule out that the prevalence of significant interaction of pitch with physiological measures stems from this choice. The absence of a systematic influence of music on the scaling of cardiac output could also derive from the smaller sample size (17/28 subjects). Finally, the DFA method applied justifiably uses a detrending procedure to mitigate the nonstationarity of the underlying processes and estimate the scaling exponent robustly. This approach may nonetheless underestimate the contribution of nonlinearities that can be meaningful in the characterisation of music and brain signals and have been shown relevant for the aesthetic appreciation of music^73^. We expect our findings can fuel research and inform motor rehabilitative tools that deploy 1/*f* auditory cueing^74^ and brain-computer interfaces that leverage acoustic and neuronal features to predict music-induced emotion^75,76^. In addition, since music induces *α*_*brain*_ decreases, maximally in occipitoparietal areas and with pleasure, and a comprehensive literature suggests this dynamical reorganisation sustains many tasks–it would be interesting to probe whether transfer effects of music (e.g., the mitigation of visual neglect in post-stroke patients by pleasant music in a visuospatial task^77^) are bolstered by these mechanisms.

To conclude, the pleasure of music derives not from the fractal structure of music dimensions *per se*, but may arise from the interaction between the acoustic scaling with the scaling behaviour of neuronal dynamics. A 1/*f resonance* between music sounds and our brain prevails when music moves us.

## Methods

### Participants

A total of 31 healthy volunteers were recruited and paid to participate in this experiment. Three participants were excluded from analysis due to technical issues during recording, which led to incomplete data. The final sample was constituted by 28 subjects (12 female, 16 male) with mean age = 26.8 ± 4.2 (SD) (27 right-handed, 1 left-handed). All participants gave written informed consent prior to the experiment. The study was performed in accordance with the guidelines approved by the Ethics Committee of the Faculty of Psychology and Education at the VU University Amsterdam. The participants had no history of neurological disease or psychiatric disorders, were not musicians and only roughly half had any formal music training. (see Supplementary Fig. S11). Only 17 out of the 28 participants underwent the ECG recording.

### Stimuli

The music excerpts (see Supplementary Table S1) were selected from a pool of piano compositions which had available scores in the Humdrum Kern database^78^; the chosen stimuli lasted at least 2 min and exceeded 200 separate note onsets^20^. The main criteria for inclusion relied on the scaling exponent of the rhythm and pitch of these compositions. In addition, the Sonata for Two Pianos in D Major K. 448 by Mozart^10^ was included. The scaling exponents (*α*) were computed based on the score using DFA (detrended fluctuation analysis)^79^ (see details below), similarly to previous studies^20,80^. Next, the pieces were binned by their values of scaling of pitch and rhythm series, and 12 compositions were selected allowing a range of values 0.5–1, trying to match compositions with similar pitch/rhythm exponents. Two distinct compositions from six different composers were chosen to integrate some stylistic diversity. Recorded performances of the selected pieces were obtained from Qobuz.com in CD-quality (lossless, 16 bits, 44.1 kHz). The stimuli were created by extracting the first 110 seconds of music from the recordings using Audacity (2.0.6), (equalizing these at a 65-dB output level (http://www.holgermitterer.eu/research.html), and adding 0.5-second long fade-in and fade-outs to prevent speaker popping—both using Praat (http://www.praat.org/).

### EEG/ECG paradigm and data acquisition

A three–minute eyes-closed rest was recorded as a baseline for the music paradigm. Both, during the rest and music listening periods, the subjects were instructed to keep their eyes closed and only open them with an auditory cue. After the period of rest and at the end of the experiment, the subjects filled the Amsterdam Resting-state Questionnaire (ARSQ) (see^81^ for details), this questionnaire data were not analysed in the present study. The music listening phase exposed the participants to 12 music excerpts with different degrees of statistical self-similarity (different *α*) regarding how its volume, melody and rhythm unfolded over time and, they were asked to rate the piece-induced level of pleasure, familiarity and concentration. The 12 music piece excerpts were presented in a randomised order. At the end, three of these pieces were played again, the same excerpts for all participants but also with randomised order. The participants were first familiarised with the task with a trial music piece, distinct from the stimuli used and instructed to adjust the volume to a comfortable level. Before each piece, a visual cue signalled that the piece would start and the subject should close their eyes and concentrate on the music until they heard a beep, after the piece’s end. A Likert-like 7-point psychometric scale was used to rate whether the subject strongly agreed (7) to strongly disagreed (1) the piece was pleasurable/familiar/easy to focus. The subjects also had to indicate whether they had opened their eyes (binary scale). Finally, the experiment ended with the filling of a questionnaire about the subject’s overall musical taste and experience (summarised results in Supplementary Fig. S11). All visual cues, instructions, and questionnaires were presented on a computer screen. Auditory cues and music stimuli were presented over KRK Rokit 8 RPG 2 studio monitors. Stimuli presentation and acquisition of behavioural data was done using custom scripts in the OpenSesame environment (v.2.8.3)^82^. EEG data were sampled at 1 kHz and the EGI Geodesic EEG system with HydroCel sensor nets consisting of 128 Ag/AgCl electrodes was used. Impedance was kept below a 50–100 kΩ range and the vertex was used as the common reference.

### EEG preprocessing

From the whole-set EEG, ten channels were excluded from analysis due to its location extrinsic to the brain. The continuous recordings were epoched in 16 segments comprising a segment of 178 seconds of eyes-closed resting state and 15 segments of 108 seconds of the different music pieces presented once or twice. The first 2 seconds of all segments were clipped while epoching (minimizing possible amplifier saturation effects when applicable). Line-noise removal followed the method for sinusoidal removal implemented in the PREP pipeline (PrepPipeline 0.55.1 see^83^). Line noise (50 Hz) and energy in its harmonics (up to 450 Hz) are preferably removed without a notch filtering so that most wide-spectral energy is preserved and only deterministic components are removed. Briefly, the method applies to a high-passed filtered version of the data, an iterative procedure to reconstruct the sinusoidal noise based on Slepian tapers (4 s, sliding window of 0.1 s), which is a posteriori removed from the original non-filtered data. Such a small sliding window ($\ll 1\,{\rm{s}}$—pipeline’s default) was crucial for line-noise removal in our data, longer windows only allowed attenuation and caused ringing artefacts. This procedure avoids high-pass or notch filters which have known pitfalls^84^, minimising distortions in the long-term structure of signals and facilitating the analysis of high-frequency EEG activity intended here. Bad channels were detected and interpolated and the robust re-referencing of the signals to an estimate of the average reference performed (all details in^83^). To mitigate transient artefacts such as myogenic/ocular activity, each segment was split into multiple sub-epochs of 0.25 *s* (a duration in which the EEG is quasi-stationary^85^) and sub-epochs with high epoch’s amplitude range, variance or difference from the epoch’s mean were not included in further analysis.

### Multiscale analysis

To study the neuronal dynamics at a multiscale level we opted to apply Empirical Mode Decomposition (EMD)^86^, a method that separates a signal *x*(*t*) in a set of *n* so-called intrinsic mode functions (IMFs)—*c*_*i*_, which represent the dynamics of the signal at different time scales:

1 $$x(t)=\mathop{\sum }\limits_{n=1}^{n}\,{c}_{i}(t)+{r}_{n}(t)$$

where *c*_*i*_(*t*) represents the *n* IMFs and *r*_*n*_(*t*) is the residue (a constant or monotonic trend). It is noteworthy that the method does not result in a separation of the signal in predetermined frequency bands but rather, in a signal-dependent time-variant filtering^87^, fully adaptive and, therefore, suitable for the nonstationary and nonlinearity of the electroencephalogram. Notwithstanding, the IMFs obtained have a defined bandwidth that can be related to the classic frequency bands used in clinical practice or neuroscientific research. Each mode has typically a power spectrum that peaks around a limited range of frequencies^87^, and a characteristic frequency given by *f*_*s*_/2^*n*+1^, where *f*_*s*_ represents the sampling frequency and *n* the number of the mode (*n* = 1, 2, 3 …). Thus, to match this filtering method with the frequency ranges of the classical bands, the first six (IMF1–IMF6) correspond to a spectral energy with peaks within roughly the range 8–250 Hz and the last three have activity around 1–4 Hz (Fig. 1 and Supplementary Fig. S1). Following the aforementioned relationship between mode number and its main frequencies, we labelled IMF1 as high-gamma (*γ*_*h*_), IMF2 as mid-gamma (*γ*_*m*_), IMF3 as low-gamma (*γ*_*l*_), IMF4 as *β*, IMF5 as *α*, IMF6 as *θ*, and finally, the sum of modes IMF7, 8 and 9 equivalent to *δ*. The method uses a *sifting* process which starts by identifying the extrema in the raw time series *x*(*t*). Next, two cubic splines are fitted, connecting the local maxima and the local minima. The average—*m*(*t*)—of these envelopes is performed and subtracted from *x*(*t*), this difference constitutes the first mode (IMF1). The residue signal (*r*_1_ = *x*(*t*) − *c*_1_) is treated as the new signal and the sifting process iterates until further modes are extracted. The optimal number of sifting is undetermined; we opted to use 10 as this choice preserves a dyadic filtering ratio across the signals^88^. The code used is available online at http://perso.ens-lyon.fr/patrick.flandrin/emd.html.

### Estimation of neuronal scaling

To assess the degree of long-range temporal correlations or equivalently the self-similarity present in the EEG segments, the instantaneous amplitudes of the fluctuations the *γ*_*h*_, *γ*_*m*_, *γ*_*l*_, *β*, *α*, *θ* and *δ* ranges were calculated using the magnitude of the analytic signals quantified using the Hilbert Transform. Next, fractal scaling exponents of these amplitude fluctuations were estimated using DFA^79^—an algorithm useful to quantify the long-range scale-free correlations in non-stationary signals, mitigating spurious detection of artefactual long-range dependency due to nonstationarity or some extrinsic trends^89^. The method is essentially a modified root mean square analysis of a random walk^30^, introduced to use in neuronal band-passed signals in^25^; full details are described elsewhere^25,30,90^. Briefly, for a given time series *x* the algorithm quantifies the relationship between *F*(*n*), the root-mean-square fluctuation of the integrated and detrended time series, and the elapsed window of time, *n*. Typically, *F*(*n*) increases with *n* and displays the asymptotic behaviour *F*(*n*) ~ *n*^*α*^. The fractal scaling exponent (*α*) was estimated by extracting the slope of a linear least-square regression of *F*(*n*) on a log-log plot within the scaling range of *n* ∈ [3, 15]. The self-similar exponent (*α*) is closely related to the auto-correlation function *C*(*τ*). When *α* = 0.5, the *C*(*τ*) is 0 for any time-lag (*τ* ≠ 0) and the signal is equivalent to white noise hence, constituting uncorrelated randomness. A scaling exponent in the range of [0.5–1] indicates the presence of persistent long-range temporal correlations and scale-free properties; the closer its value is to one, the greatest its self-similarity—in this case *C*(*τ*) ~ *τ*^*γ*^ where *γ* = 2 − 2*α*. Within this regime, there is a straightforward relationship between the auto-correlation and the power spectral density, *P*(*f*). Following the Wiener-Khintchine theorem, considering that *P*(*f*) ~ 1/*f*^*β*^ and *β* = 1 − *γ*, thus *β* = 2*α* − 1. Above one (*α* > 1), the underlying signal still displays long-range temporal correlations but these are not temporally structured in a fractal manner. The same bounds of the fitting range (3 and 15 *s*) were used in the estimation of *α* from all resting state or music listening segments. Scaling behaviour analysis was based on adapted functions from the Neurophysiological Biomarker Toolbox (NBT v.0.6.5–alpha, www.nbtwiki.net)^90^, in MATLAB (R2015a; The MathWorks, Inc).

### Analysis of musical dimensions

Music stems from the orderly sequencing of sounds; while adding notes or increasing the volume does not produce music, the manipulation of sound’s intensity, of its melody (successive changes in pitch) and rhythm (successive changes in tone duration), contribute essentially to musical meaning^19,80^. To obtain estimates of the loudness, pitch and rhythm variation embedded in the stimuli presented we used the MATLAB-based MIR toolbox (v.1.7)^91^. We obtained these dimensions from the 110 s stimuli audio recordings to ideally quantify the exact scaling properties of the excerpts the subjects were exposed to—performers can vary in their interpretations of music scores and a segment’s scaling properties can diverge from those of the whole piece. To approximate the acoustic amplitude and frequency properties of the music pieces to the perceived loudness and pitch, we used as an auditory model the 2-channel filterbank developed in^92^, composed of 2-channels (one above and the other below 1 kHz). Estimations were performed in a band-wise fashion and the results were later merged. To estimate loudness, the spectrogram of the excerpts was obtained with a window size of 0.1 *s*, Hann windowing with 10% overlap; the envelope was extracted after 40 Hz high-pass filtering with the Butterworth filter implemented in the toolbox, half-wave rectified and down-sampled to 2756.25 Hz. For pitch calculation, the several notes/chords need to be detected at a given instant; the polyphony, which arises from notes being played simultaneously by distinct hands, challenges accurate pitch tracking due to masking effects^93^. We found that using the mirpitch() function with default settings in the toolbox performed nearly optimally; for the notes in each frame, a dominant pitch/fundamental frequency was extracted. This resulted in a monodic curve sampled at 100 Hz to which we applied the first derivative to obtain the pitch successions. To estimate the rhythm, a similar procedure was followed to obtain the times of the note onsets using the default threshold of 0.4. The so-called novelty curve—which captures sudden changes in the music signal by means of peaks that represent onset candidates—was obtained by applying the “spectral flux” implemented in the mironsets() function, a widely used method based on the signal short-time spectrum^93^. The differences in time between notes were taken as the rhythm series.

The estimation of the scaling exponent of the musical dimensions was done as described for the neuronal activity, in the same range of 3–15 seconds for best comparison to the neuronal scaling. The average across the 12 music stimuli yielded the following median dimension estimates: 1.07 ± 0.14 (loudness), 0.88 ± 0.10 (pitch) and 0.72 ± 0.13 (rhythm). An additional estimate of the overall scaling of music was taken by averaging the scaling exponent of the three dimensions.

### Spectral power estimation of neuronal activity

As a control to the data quality, the averaged spectral power was also estimated in seven frequency bands *δ* (1–3 Hz), *θ* (4–7 Hz), *α* (8–12 Hz), *β* (13–30 Hz), *γ*_*l*_ (31–45 Hz), *γ*_*m*_ (55–125 Hz) and *γ*_*h*_ (126–345 Hz)) by applying the Welch’s modified periodogram method implemented in Matlab’s *pwelch()* function with 50% overlapping Hann windows of 2.048 seconds, the averaged power was computed by integrating the power spectral densities within each band and calculating the mean.

### ECG analysis

The ECG data were sampled at 1 kHz, a low-pass filter (cut-off frequency: 100 Hz) and a notch filter (that attenuates frequencies around 50 Hz, default from the acquisition system) were applied during recording. After acquisition, high-pass and low-pass two-way constrained least squares FIR filters (cutoff 45 Hz, order = 500; cutoff 0.5 Hz, order = 1200, −70 dB stopband attenuation, passband ripple = 0.1%) were used to eliminate baseline drift of non-cardiac origin and minimize other artifacts such as power line interference or electromyographic noise. To study heart rate variability (HRV), the distances between sinus beats (so-called RR or interbeat intervals) were computed from the preprocessed signals of the subjects (*n* = 17). Ectopic beats were eliminated automatically by excluding the beats deviating >20% from the previous one and also by visual inspection. In most cases, the automatic procedure resulted in 100% or close to 100% of the beats being kept; however, for a few segments this value approached 70–90% and, therefore, we opted to not use any method for linear interpolation of the excluded beats^94^. Following visual inspection, 9/272 segments had data removed due to artefacts. With the cleaned interbeat series (NN intervals), the scaling exponent or self-similarity of the cardiac rate variability was estimated in a range between 4 and 11 beats. The procedure was similar to the described for EEG time series. Of note, this value is considered an index of short-term heart-rate variability and we will designate it here as *α*_1_ (*heart*)^30^, to distinguish from the fractal scaling often computed on longer timescales. The range of ~4–11 beats coincides with the interval 3–15 seconds used in the computation of the neuronal and musical scaling parameters if the heart rate is close to normal baseline levels (~60 bpm) and is slightly shorter if heart rate accelerates beyond that level. The following standard measures of short-term HRV were also calculated from the NN time intervals: AVNN (average heart rate), SDNN (standard deviation), rMSSD (square root of the mean of the squares of differences between adjacent NN intervals), pNN50 (percentage of NN >50 ms) and the frequency domain measures—VLF (spectral power of the NN interval time series between 0.003 and 0.04 Hz), LF(spectral power of the NN interval time series between 0.04 and 0.15 Hz), HF (spectral power of the NN interval time series between 0.15 and 0.4 Hz), and LF/HF (ratio of low to high frequency). The frequency domain measures were computed using the Lomb-Scargle periodogram method. The analysis were performed utilizing the PhysioNet Cardiovascular Signal Toolbox^94^ available at www.physionet.org ^95^.

### Statistical testing

To study the overall effect of music in ongoing neuronal dynamics, we averaged *α*_*brain*_ of the several timescales [*γ*_*h*_, *γ*_*m*_, *γ*_*l*_ …] across the 12 music pieces firstly presented and compared to the values during resting-state using Wilcoxon signed rank tests (*p* < 0.05, two-tailed), in light of the null hypothesis that the scaling behaviour is identical in these two states. Spearman correlation was applied to associate these changes in neuronal dynamics with the individual’s behavioural score, due to its nonparametric character and also the ordinal nature of the psychometric scoring^96^. We also investigated the interaction between the music and the neuronal dynamics: averages of *α*_*brain*_ for each component [*γ*_*h*_, *γ*_*m*_, *γ*_*l*_ …], across the *n* = 28 subjects for each piece of music were plotted against the scaling exponent (of each music dimension: *α*_*loudness*_, *α*_*pitch*_ and *α*_*rhythm*_) of those same pieces and the Spearman’s correlation coefficient (*r*_*s*_) computed. Similarly, associations between the scaling behaviour of music and HRV indices were computed for the subset of subjects with recorded ECG (*n* = 17). To correct for multiple testing associated with the 119 channel locations, Type I errors were minimised by employing a false discovery rate (FDR) correction^97^, the FDR threshold was set so that 5%, 10% and 20% of the supra-threshold differences or correlations are expected to be false positives and the results compared across these levels of statistical significance.

Head surfaces displayed were created by adapting the *headplot* Matlab code from EEGLab https://sccn.ucsd.edu/eeglab/ ^98^. For visualisation purposes, a locally weighted regression smooth line was added to scatterplots by using the *loess* fit^99^ implemented in R^100^ with various smoothing spans and a polynomial degree of 2. The *loess* fit is suitable as a nonparametric descriptive of the monotonic associations reported; no steps were taken to ensure the monotonicity of the bivariate relationship but, it was present in most cases. Importantly, the associations seldom approached a linear relationship, justifying the need for nonparametric statistical testing and fitting.

## Supplementary information

Supplementary Information

## Data Availability

The datasets generated and analysed during the current study are available from the corresponding author on reasonable request.

## Code availability

Any custom code used during the current study is available from the corresponding author on reasonable request.
