# Supplementary material for: Scaling behaviour in music and cortical dynamics interplay to mediate music listening pleasure
Source: Sci Rep. 2019 Nov 27;9:17700. doi: 10.1038/s41598-019-54060-x (PMC6881362; doi:10.1038/s41598-019-54060-x)
Supplement: Supplementary file 1 — Supplementary Information [file 41598_2019_54060_MOESM1_ESM.pdf]

Scaling behaviour in music and cortical dynamics interplay to mediate music listening pleasure

Ana Filipa Teixeira Borges[a,*], Mona Irrmischer[a], Thomas Brockmeier[a], Dirk J.A. Smit[b], Huibert D. Mansvelder[a], Klaus Linkenkaer-Hansen[a]

a Department of Integrative Neurophysiology, Center for Neurogenomics and Cognitive Research (CNCR), Amsterdam Neuroscience, VU Amsterdam, Amsterdam,1081 HV, Netherlands
b Psychiatry department, Amsterdam Neuroscience, Academic Medical Center, University of Amsterdam, 1081 HJ Amsterdam, The Netherlands

*** aftborges@gmail.com**

Supplementary Results

Global changes in neuronal scaling behaviour

On a global topographic level, the median scaling of neuronal activity decreased monotonically from the highest-frequency, γ_h, (scaling exponent: $\alpha \approx 1$) to the lowest (θ) (scaling exponent, $\alpha \approx 0.65$); except for δ , which also exhibited an $\alpha \approx 1$) (Supplementary Fig. 2). Although this pattern was consistent across rest and music listening, the neuronal scaling exponents of activity from β to θ were reduced and increased in higher-frequency amplitude modulations ($\geqslant \gamma_m$) during music in comparison to rest; see statistical significance details in Supplementary Table 2 and Supplementary Fig. 2.

Association of Familiarity to the music with neuronal scaling

To establish the interaction between the familiarity of the music pieces with the neuronal scaling behaviour, we correlated the individual average score of Familiarity ($\bar{s}_{individual}$) with the α_{brain} in $[\gamma_h, \gamma_m, \gamma_l, ...]$. We observed significant associations only during music listening (and not during rest). Specifically, the scaling exponents in the frontal (α- and β-bands) and occipitoparietal (β) regions were inversely correlated to individual level of familiarity to the pieces ($q = 0.1$, FDR, see Supplementary Fig. 6 for general topographies and statistical significance details). Familiarity was also the only behavioural measure significantly associated with the scaling of the music signals

(inverse correlation). Concomitantly, the scaling of the music (α_{music}) was found to correlate with the α_{brain} (Fig. 4, Supplementary Fig. 7) therefore, it is possible that the association Familiarity/α_{brain} stems from the influenced exerted by the physical properties of the stimuli (α_{music}) on the neuronal scaling behaviour. This hypothesis is strengthened by the lack of a significant association Familiarity/α_{brain} during resting-state.

Supplementary Figures

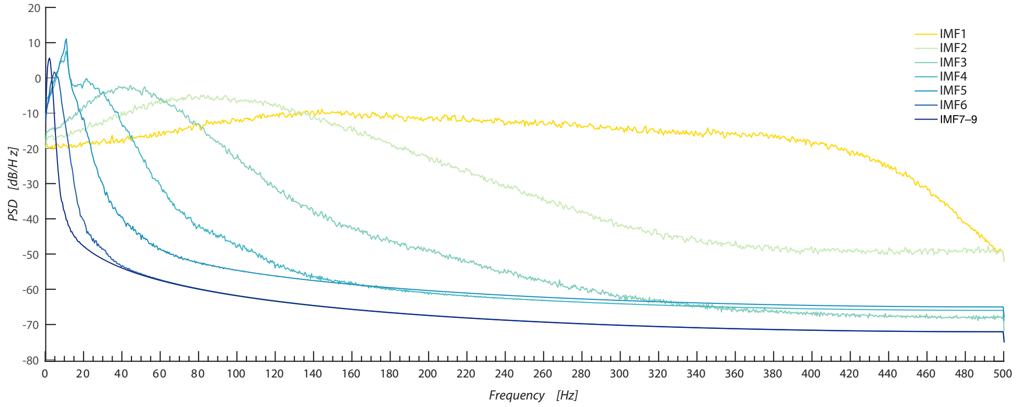

Figure S1. Distributions of spectral power of the intrinsic modes studied (IMFs) reveal how the method is similar to a dyadic filtering with the modes displaying characteristic frequencies $f_s/2^{n+1}$, where f_s represents the sampling frequency and n the number of the mode. Plots show the power spectral densities for each of the modes or group of modes used as a function of frequency from the EEG recording (occipital channel) during listening of a music piece.

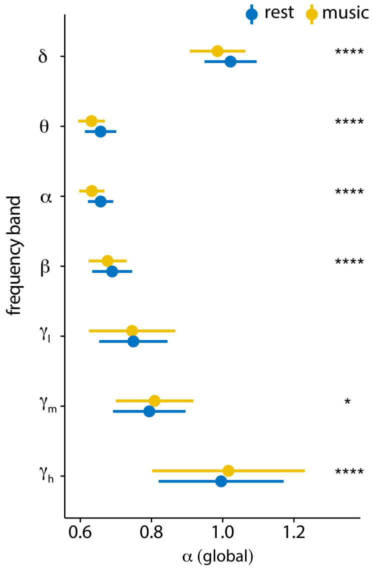

Figure S2. Neuronal scaling of the overall scalp topography during rest and music listening shows a timescale-dependent pattern. Global median scaling exponents during rest (blue) and music (yellow) for the frequency bands. The lines represent median absolute deviation. Significant statistical differences (Wilcoxon signed rank test) are represented by the stars (*, *Right*) * corresponds to $p \leq 0.05$, ** to $p \leq 0.01$, *** to $p \leq 0.001$ and **** to $p \leq 0.000$.

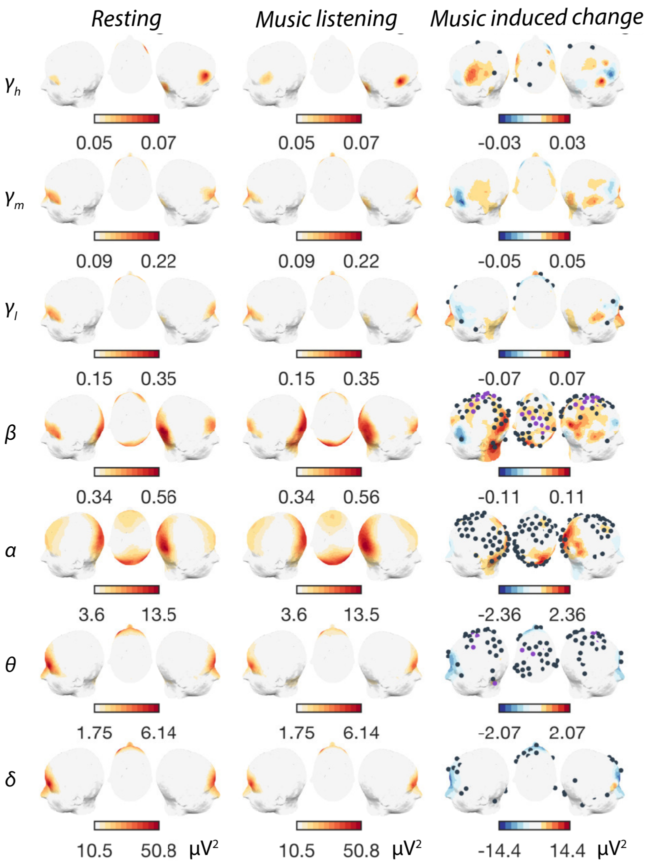

Figure S3. Spectral power changes induced by the music. Head surface maps of the spectral power in the frequency bands (γ_h–δ) during rest, during a music listening task, and of the difference of power between the two conditions. Channels marked with dark blue dots display significant differences ($p < 0.05$, uncorrected), purple dots signal significant differences after FDR correction ($q = 0.05$).

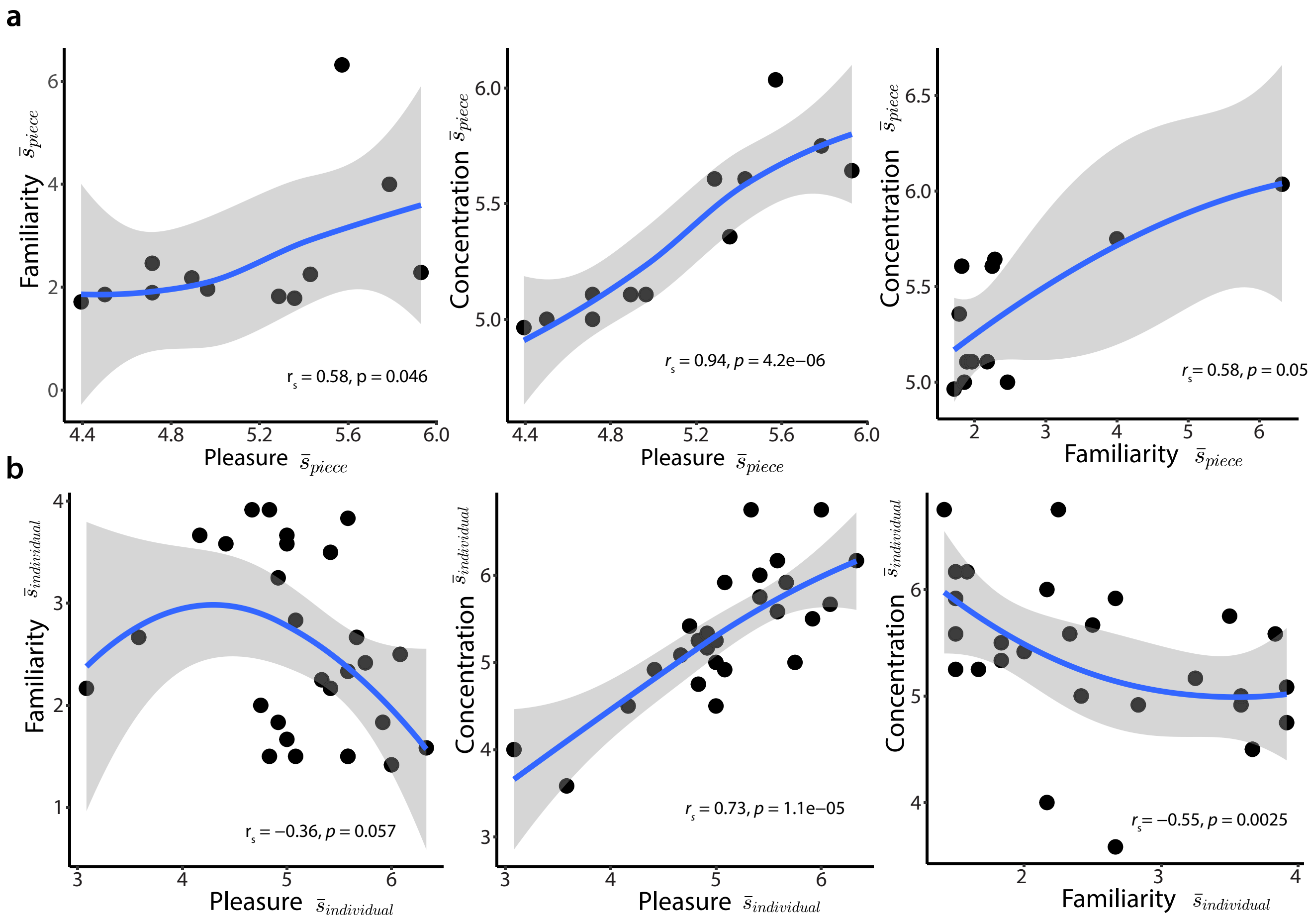

Figure S4. Interdependency between the measures of behaviour analysed. Pleasure and Concentration are strongly associated. (a) Scatterplots portray the relationship between the average score ($n = 28$) of Pleasure, Familiarity and Concentration for each piece. (b) Idem but highlighting the correlation of the average individual scores across the 12 music pieces. The blue lines represent a locally weighted regression line and the shadowed area the confidence interval and r_s the Spearman correlation coefficient.

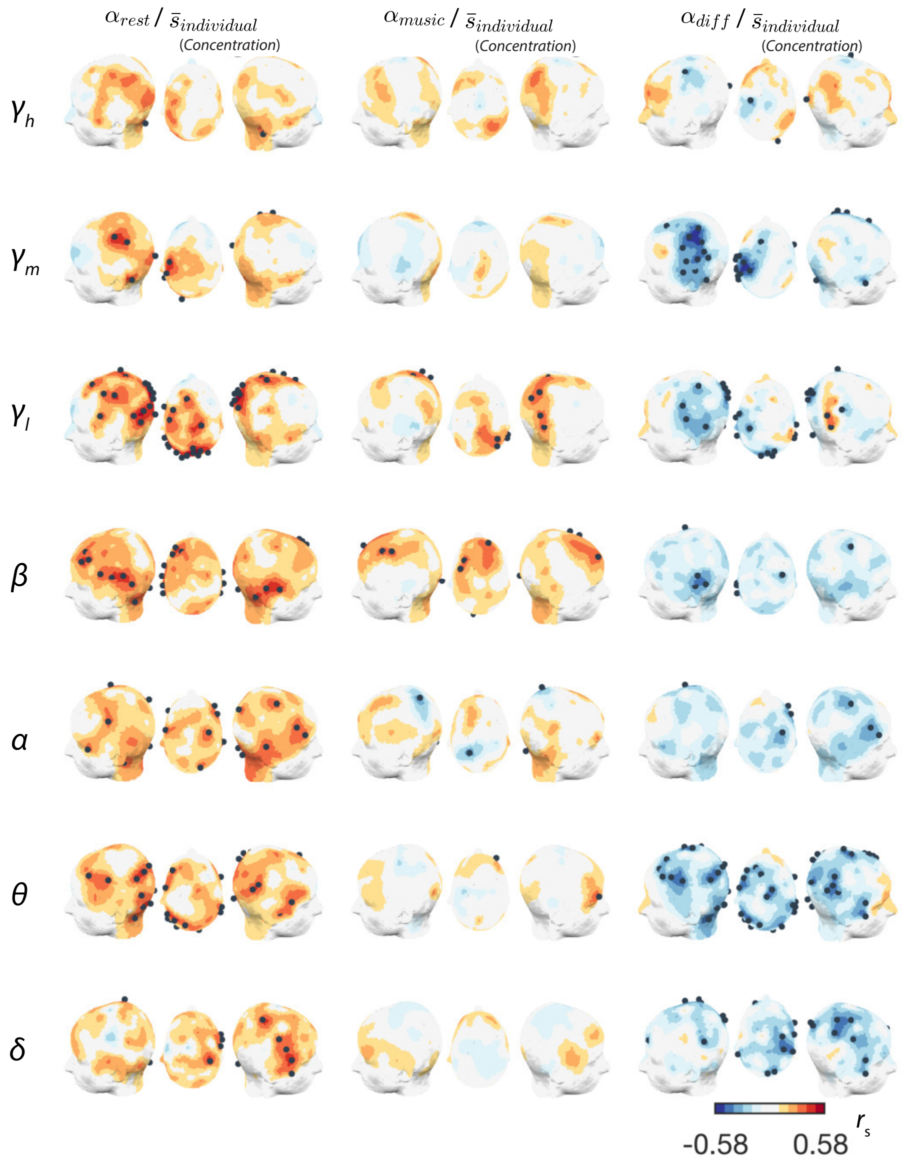

Figure S5. Association between neuronal scaling behaviour in distinct frequency bands and individual overall concentration during music listening. Head surface mappings of the correlations (Spearman coefficient—r_s) between Concentration and the scaling exponents of the frequency bands (γ_h-δ) during baseline (rest), during a music listening task, and with the induced change in scaling between the latter. The channels marked in dark blue indicate a significant correlation (p <0.05), purple dots would show significant correlations after FDR correction, which are in this case inexistent.

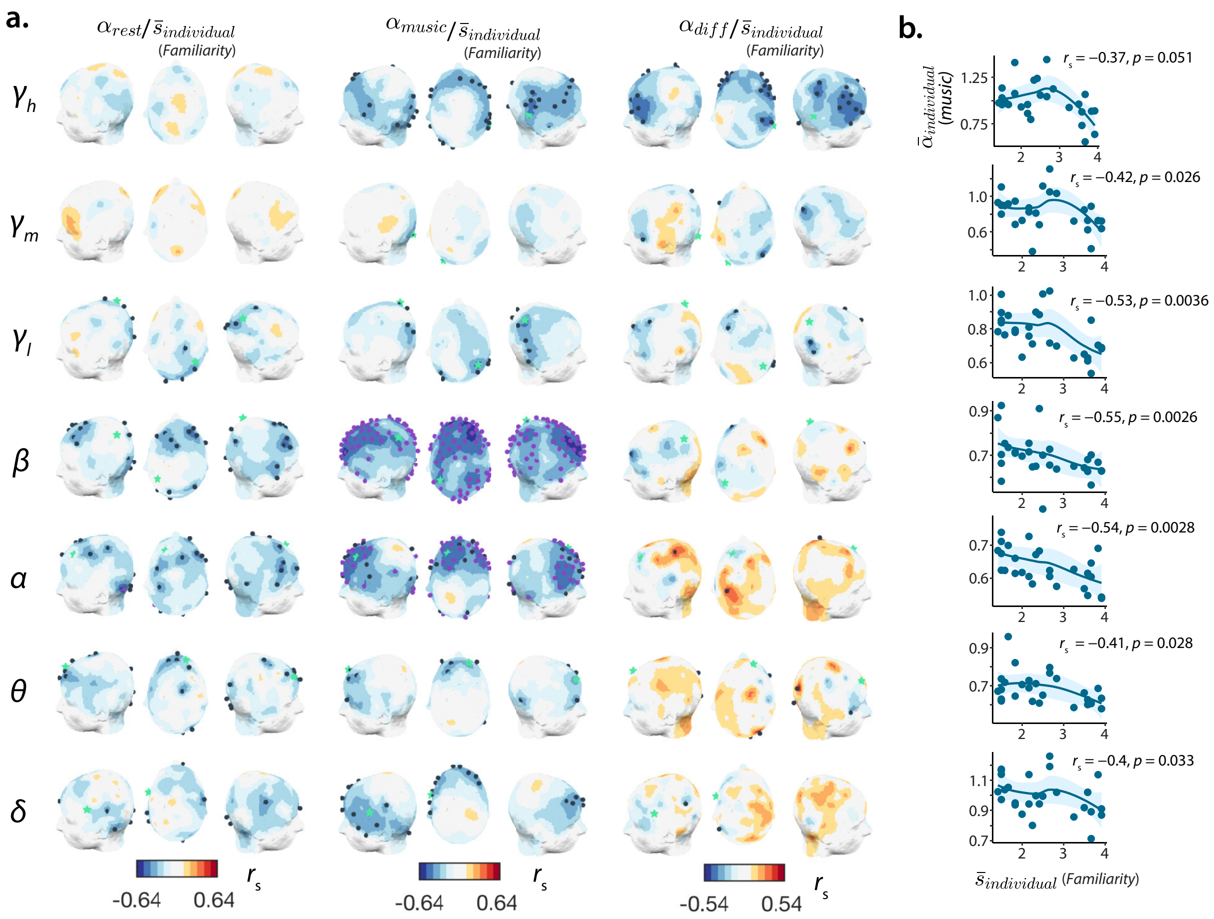

Figure S6. Individual Familiarity to the music pieces is associated to lower scaling exponents prominently in α/β neuronal activity. (a) Head surface mappings of the associations between individual Familiarity and the scaling exponents of the frequency bands (γ_h—δ) during baseline (rest), during a music listening task, and with the induced change in scaling between the latter. The channels marked in dark blue indicate a significant correlation (Spearman coefficient r_s, $p < 0.05$), the channels in purple show significant association after FDR correction ($q = 0.1$). (b) Scatter plots of the highlighted channels (green star in (a) exemplify the individual ($n = 28$) associations, a locally weighted regression line was added to aid visualising the relationship and the shadowed area represent the confidence interval.

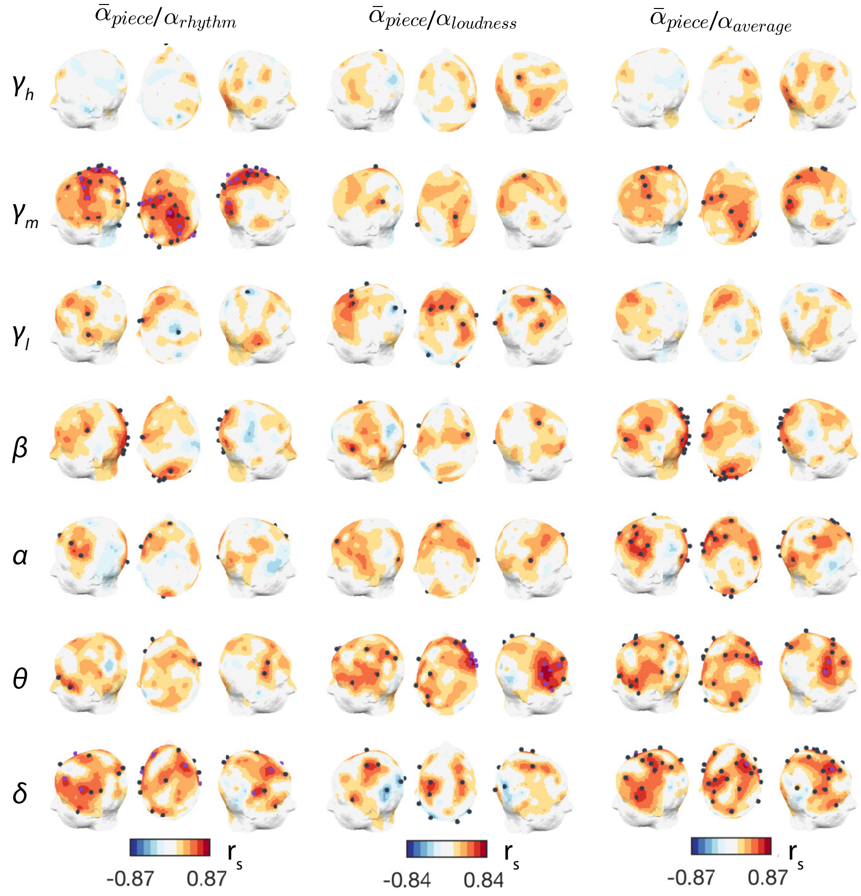

Figure S7. Association between the scaling of neuronal activity and the scaling of rhythm, loudness and averaged scaling of the music pieces. Headplots of the distribution of correlation coefficients between the scaling exponent of the rhythm, loudness and averaged musical dimension time series (α_{rhythm}, $\alpha_{loudness}$, $\alpha_{average}$) and the scaling exhibited by the neuronal activity ($\bar{\alpha}_{piece}$)—frequency bands from γ_h—δ; The channels in dark blue indicate a significant correlation (Spearman coefficient r_s, p $<$ 0.05),the channels in purple show significant association after FDR correction (q=0.2, α_{rhythm}— minimum p=0.0133 (γ_m) and p=0.0032 (δ); $\alpha_{loudness}$ — minimum p=0.0066 (θ); average — minimum p=0.0014 (θ) and $p = 5.98 \times 10^{-4}$ (δ)).

a

Music pieces	Estimated average tempo (excerpt) [bpm]	Tempo marking (classical description–entire piece)
Ludwig von Beethoven 1	121.83	andante
Ludwig von Beethoven 2	94.18	allegro vivace
Johannes Brahms 1	59.84	andante
Johannes Brahms 2	99.00	andante
Frederic Chopin 1	82.48	andantino
Frederic Chopin 2	102.86	andante
Edvard Grieg 1	131.12	tempo di Mazurka
Edvard Grieg 2	164.55	speeds up until prestissimo finale
Joseph Haydn 1	158.67	presto
Joseph Haydn 2	85.14	allegro
Wolfgang Amadeus Mozart 1	107.05	andante cantabile
Wolfgang Amadeus Mozart 2	141.29	allegro con spirito

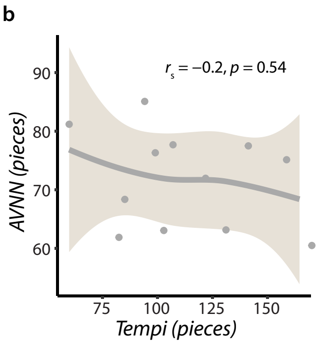

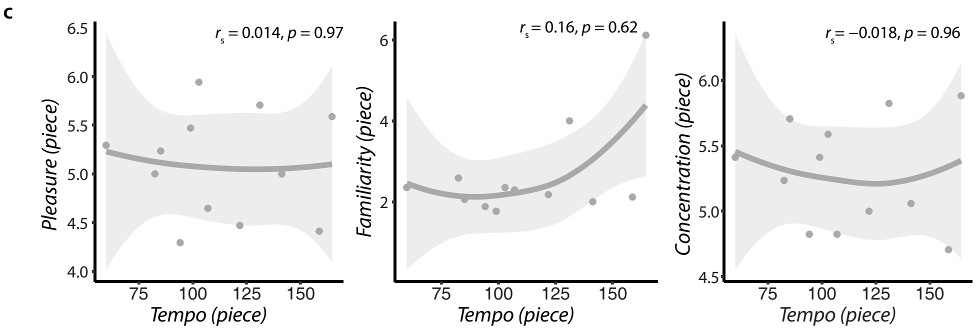

Figure S8. The tempi of the music piece's excerpts has no effect on averaged heart rate or behaviour measures during music listening.(a) List of music pieces codes, respective averaged tempo computed from the music excerpts and classical tempo marking associated with the pieces.(b) Relationship (*ns*) between the average heart rate (AVNN) and the tempi estimated (a); r_s denotes the Spearman correlation coefficient.(c) Similar to (b) showing the relationship to the averaged behaviour measures of each piece excerpt.

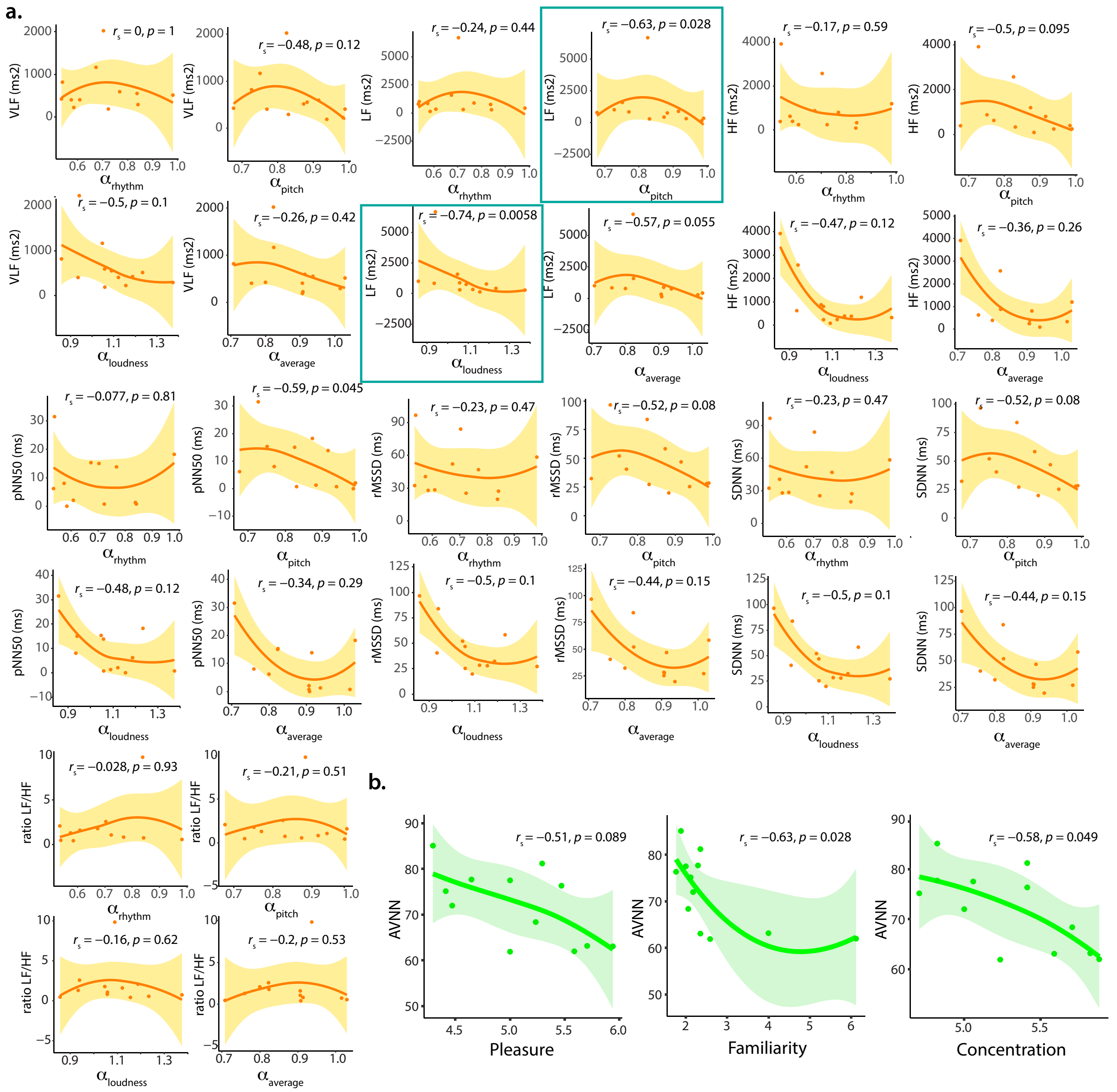

Figure S9. Relationship between traditional short-term heart rate variability indices and the scaling of musical dimensions/behaviour measures. (a) Scatterplots of the frequency- (VLF, LF, HF and LF/HF) and time-domain measures (pNN50, rMSSD and SDNN) vs. α_{rhythm}, α_{pitch}, $\alpha_{loudness}$ and $\alpha_{average}$. (See Methods for full details on measures) (b) Scatterplots of the individual average heart rate (AVNN) vs. the averaged individual behaviour scores. The lines represent a locally weighted regression line and the shadowed the confidence interval, r_s the Spearman correlation coefficient.

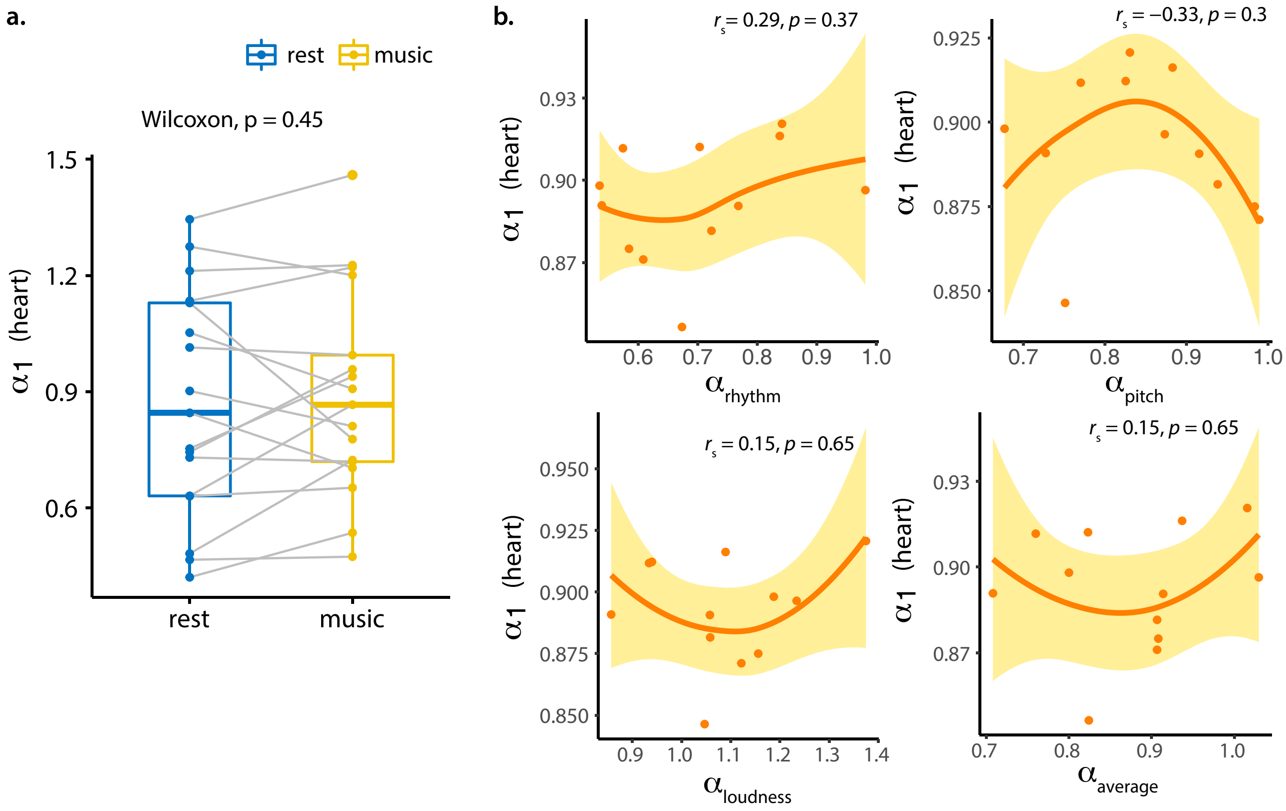

Figure S10. Scaling of heart-rate dynamics displays no systematic effect of music listening and of the scaling of music loudness, pitch and rhythm. (a) Boxplots of the scaling of individual (n=17) heart rate variability (α_1) during music listening vs. baseline rest. (b) Scatterplots showing the association (ns) between α_1 and the scaling of each of the musical dimensions (α_{rhythm}, α_{pitch} and $\alpha_{loudness}$) and the averaged scaling between the three dimensions ($\alpha_{average}$).

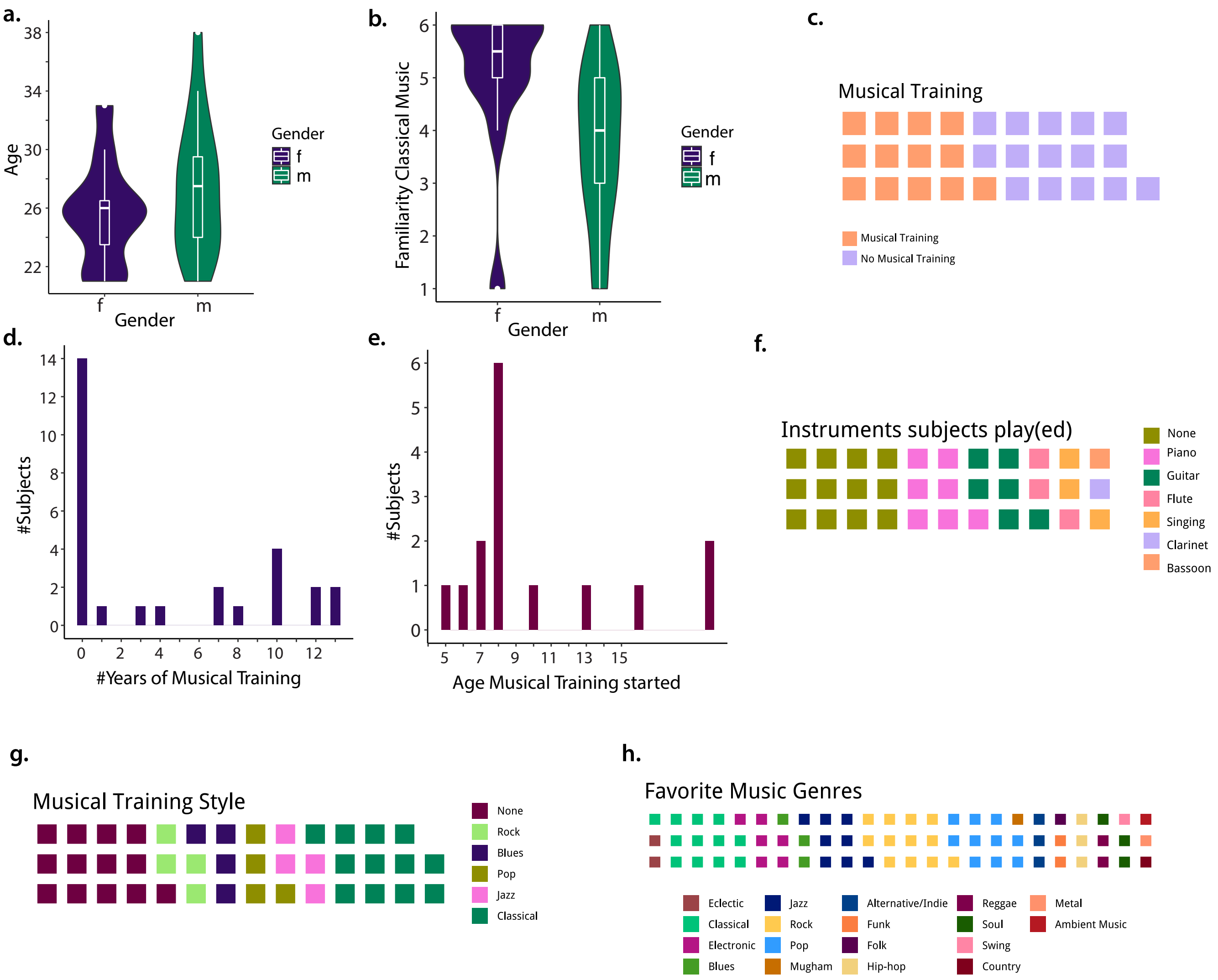

Figure S11. Overview of additional participant information data. (a) Violin plots of the age of the participants split by gender overlaid by boxplots with whiskers that extend 1.5 times the interquartile range. (b) Idem for the distributions of reported Familiarity with Classical Music assessed on a 7-point Likert-like scale. (c) Waffle chart of the number of participants with (no) formal musical training. (d) Histogram of the number of participants by length of musical training. (e) Histogram of the number of participants by age of start of the musical training. (f) Waffle chart of the proportion of subjects that play (g) Idem for the number of subjects that undergo different music styles. (h) Idem for the number of participants that indicate a music genre as a favourite. In all waffle charts each square represents one participant.

Supplementary Tables

27

Table 1. List of the 12 music pieces used in the experiment.

Composer	Performer	Piece
Ludwig von Beethoven	Abdel Rahman El Bacha	Piano Sonata No. 13 in E-Flat Major, Op. 27 No. 1 'Sonata quasi una fantasia': 1. Andante - Allegro - Andante
Ludwig von Beethoven	François-Frédéric Guy	Piano Sonata No. 15 in D Major, Op. 28 'Pastorale': 3. Scherzo (Allegro vivace)
Johannes Brahms	Alexander Melnikov	Piano Sonata in C major, Op.1: Andante (nach einem altdeutschen Minnelied)
Johannes Brahms	Maurizio Pollini	Scherzo in E-flat Minor, Op.4
Frederic Chopin	Janina Fialkowska	Mazurka No. 49 in F Minor, Op. 68, No. 4
Frederic Chopin	François Chaplin	Nocturne in E Minor, Op. 72 No. 1, Op. Posthume
Edvard Grieg	Claudio Colombo	Peer Gynt, Suite No. 1 Op. 46 for Piano: 3. Anitra's Dance
Edvard Grieg	Claudio Colombo	Peer Gynt, Suite No. 1 Op. 46 for Piano: 4. In The Hall Of The Mountain King
Joseph Haydn	Ekaterina Derzhavina	Keyboard Sonata (Partita) No. 16 in D Major, Hob.XVI:14: 3. Finale: Presto
Joseph Haydn	Sviatoslav Richter	Piano Sonata in E-Flat Major, Hob.XVI:.52: 1. Allegro
Wolfgang Amadeus Mozart	Mitsuko Uchida	Piano Sonata No.10 in C Major, K.330: 2. Andante cantabile
Wolfgang Amadeus Mozart	Güher Süher Pekinel	Sonata for Piano Duet in D Major, K448 : 1. Allegro con spirito

Table 2. Global median scaling exponents during rest and music and respective Wilcoxon signed rank sum test statistic, z-score and size effect (r) of the difference between the two conditions for all the modes/frequency bands.

Intrinsic mode(s)–EMD decomposition	Frequency band	Mdn Rest	Mdn Music	W	z	r
1	γ_h	0.9956	1.0161	234	0.71	0.09
2	γ_m	0.7937	0.8087	117	-1.96	-0.26
3	γ_l	0.7490	0.7455	196	-0.16	-0.02
4	β	0.6895	0.6765	91	-2.55	-0.34
5	α	0.6571	0.6323	78	-2.85	-0.38
6	θ	0.6569	0.6314	130	-1.66	-0.22
7–9	δ	1.0220	0.9855	99	-2.37	-0.32
